# A Narrative Review of Childhood Picky Eating and Its Relationship to Food Intakes, Nutritional Status, and Growth

**DOI:** 10.3390/nu10121992

**Published:** 2018-12-15

**Authors:** Tinu Mary Samuel, Kathy Musa-Veloso, Manki Ho, Carolina Venditti, Yassaman Shahkhalili-Dulloo

**Affiliations:** 1 Nestle Institute of Health Science, Nestle Research, Route du Jorat 57, Vers-chez-les-Blanc, 1000 Lausanne, Switzerland; TinuMary.Samuel@rdls.nestle.com; 2 Intertek Health Sciences Inc., Food and Nutrition Group, 2233 Argentia Road, Suite 201, Mississauga, ON L5N 2X7, Canada; kathy.musa-veloso@intertek.com (K.M.-V.); manki.ho@intertek.com (M.H.); carolina.venditti@intertek.com (C.V.)

**Keywords:** picky eating, food intake, diet, macronutrient, micronutrient, growth, children

## Abstract

A main characteristic of children perceived as picky eaters is their tendency to avoid certain foods or food groups. The goal of this narrative review is to provide an overview of published studies that have examined whether picky eating in childhood is in fact associated with measurable differences in food and/or nutrient intakes and growth. While picky eaters appear to consume less vegetables compared to non-picky eaters, no consistent differences were observed for the intakes of other food groups or the intakes of energy, macronutrients and dietary fiber. Although, in some studies, picky eaters had lower intakes of certain vitamins and minerals, the levels consumed generally exceeded the recommended values, suggesting nutritional requirements are being met. No consistent relationship between childhood picky eating and growth status was observed, although significant differences in body weight/growth between picky and non-picky eaters were most discernible in studies where multiple defining criteria were used to identify picky eating. The research area would benefit from the adoption of a uniform definition of picky eating. More longitudinal assessments are also required to understand the long-term impact of picky eating on nutritional status and growth.

## 1. Introduction

Picky eating, which is also referred to as fussy eating, selective eating, faddy eating, and choosy eating, is a complex behavior that broadly refers to a combination of traits. As children start complementary feeding and become exposed to an increasingly diversified diet, many begin to exhibit “picky eating” behaviors. Currently, there is no concise definition for picky eating that has been widely adopted in the literature. Instead, as noted by Jacobi et al. [1], picky eating has been described as “*more of an umbrella term for a spectrum of characteristics perceived by a caretaker or researcher*”.

Some of the common behavioral traits that have been used to characterize picky eating include food selectivity (i.e., avoiding the intake of certain foods or food groups), sensory-sensitivity (i.e., avoidance of a food based on its sensory properties, or requiring the preparation or presentation of meals in a very particular way) and lack of interest in eating (i.e., eats only small amounts of food, has a poor appetite, eats slowly) [1,2,3,4,5,6,7,8]. For instance, when asked to openly describe their child’s eating behaviors, many parents of children perceived to be picky eaters reported that they tend to prefer eating foods predominantly from one food group (e.g., “breads only” or “fruits only”), or to avoid certain food groups altogether (e.g., vegetables) [3,4,9]. Picky eaters may also eat only a limited number of items from each food group (e.g., “eats only cereal or waffles from the bread group”, or “chicken nuggets only from the meat group”, or “won’t eat any meat except turkey”) [3,4,9]. Additionally, parents of picky eaters are more likely to report that their child does not consume an adequate amount of food at each meal [4,8], or that the child does not eat the amount of food that the parent thinks they should be eating [3,9]. In some cases, children who are viewed as picky eaters may also exhibit food neophobia (i.e., an unwillingness to try new and unfamiliar foods) [3,4].

It is well recognized that a nutritionally-balanced diet is critical for ensuring normal growth and development in children. Current dietary guidelines for children promote the intake of a varied diet filled with healthy, nutrient-dense foods, comprising a wide range of vegetables and fruits, cereals (preferably whole grains), lean proteins, and low-fat dairy products [10,11]. Foods that are high in saturated fat, added sugars, and added salt, which could displace the intake of healthier alternatives, should be limited [10,11]. These dietary recommendations help to ensure the adequate intake of nutrients that are considered necessary for the proper growth and development of children. The variety of foods consumed is also important, given that individual foods within each food group can differ with respect to their nutritional profiles. For example, within the protein group, poultry and meats provide rich sources of niacin and zinc, while seafoods are rich in omega-3 long-chain polyunsaturated fatty acids, eicosapentaenoic acid and docosahexaenoic acid [10]. Meat, poultry and seafoods are also sources of heme iron, which is more bioavailable than the non-heme iron present in plant proteins [10].

Even though picky eating is often viewed as a common and normal part of a child’s growth and development (i.e., from the time solid foods are introduced to early childhood), such selective eating behaviors may lead to a limited intake of certain foods or food groups, and accordingly, of key nutrients. In some children, it is possible that such disturbances in eating behaviors may result in a failure to meet adequate nutritional and/or energy needs, which could have serious and negative implications on health (e.g., growth impediment, nutritional deficiency, or other functional impairments). Indeed, for a parent or caregiver, the picky eating behaviors of the child can be quite worrisome. It is important to understand whether perceptions of picky eating are indeed associated with reduced diet variety and nutrient intakes, and if these, in turn, have any implications on the child’s nutritional status and growth.

Despite the lack of a “gold-standard” definition or tool available for identifying picky eaters, many studies have been published in recent years on the subject. These include studies that assessed whether picky eating, which is often perceived by the parent or caregiver based on their child’s selective eating behaviors and limited food choices, is, in fact, consistently associated with measurable differences in food intake.

In this narrative review, studies that assessed the food preferences and/or intakes of energy, macronutrients, and micronutrients of children perceived as picky eaters (e.g., as collected through food records), or measures of growth, are presented to better understand the nutritional and clinical consequences of picky eating among children.

## 2. Methods

A search of the scientific literature was conducted to identify pertinent publications on picky eating in children that were full-length peer-reviewed studies, published between 1 January 1950 and 31 July 2017. Eleven literature databases (Adis Clinical Trials Insight, Allied & Complementary Medicine™, BIOSIS Previews^®^, CAB ABSTRACTS, Embase^®^, Foodline^®^: SCIENCE, FSTA^®^, Gale Group Health Periodicals Database, Global Health, MEDLINE^®^, and NTIS: National Technical Information Service) were searched using the electronic search tool ProQuest Dialog™. PubMed and Google Scholar were also searched. The search terms were selected to broadly identify any publications related to picky eating or its commonly used synonyms, such as food pickiness, fussy eating, finicky eating, choosy eating, selective eating, food refusal, and faddy eating. Additionally, terms related to children (i.e., infant, baby, toddler, children, boy, girl, teen, adolescent, youth, kid, preschool, youngster, or tot), and variations of such terms, were used to stratify the publication search more fully. It should be noted that subjects in each of the included studies were generally healthy (i.e., studies wherein the research was conducted on subjects with a disease or medical condition for which secondary interventions were required for feeding were not included in this review). Studies that involved children with formally diagnosed eating disorders were also not included in this review. Over 3000 publication titles, both quantitative and qualitative in nature, were screened for their relevance to this narrative review. For the purposes of this review, a publication was deemed to be relevant for inclusion if it assessed the relationship between picky eating and dietary intakes (i.e., intakes of foods/food groups, energy, macronutrients, and/or micronutrients) or measures of growth in children. Only articles with full texts available in English were considered in this review.

The information that was extracted from each article included the study design, country of conduct, the demographics of the studied population (number of children, gender distribution, mean age at study entry), how picky eating was diagnosed/defined, the prevalence of picky eating, and the method of dietary intake assessment (if relevant). Food/food group preferences and nutritional intakes (i.e., of energy, macronutrients, vitamins, and minerals) were compared between picky and non-picky eaters. Additionally, the growth/body weight status of picky and non-picky eaters was compared.

## 3. Results

### 3.1. Overview of the Identified Studies

A total of 38 publications were identified in which the effects of picky eating on food preferences, nutritional intakes, or growth/body weight were assessed. An overview of the publications is provided in Table 1. As can be seen in Table 1, the studies have been grouped according to the type of tool that was used in the classification of children as picky versus non-picky eaters; the research is dependent on the classification system employed. The tools were categorized into one of three categories; namely: (i) Use of a single closed-ended question with a Yes/No response to the question, “Is your child a picky eater?”; (ii) Use of a single question with a response selected from several possible pre-established responses; or (iii) Use of several questions and a combination of responses.

A single closed-ended question was used to determine whether the child was or was not a picky eater in three studies [3,9,12]. In these three studies, the mean age of the children ranged from 6 to 36 months, and the prevalence of picky eating ranged from 12.3 to 49%.

In 14 studies, a single question with pre-defined responses was used to establish whether the child was a picky eater [1,5,6,7,8,13,14,15,16,17,18,19,20,21]. Although a similar question (i.e., “Is your child a picky eater”) was used in several of these studies to identify children who were picky eaters, the frequency at which the question was asked, and the cut-offs used to identify picky eaters, differed across the studies, even across studies conducted by the same research group. For example, in an earlier study by Jacobi et al. [5], the question “Is your child a picky eater?” was used to identify children who were picky eaters; the question was asked at two different interviews, and to be classified as a picky eater, a score of at least 3 (“sometimes”) at one of the two interviews and a score of at least 4 (“often”) at the other interview were required. In contrast, in a later study by Jacobi et al. [1], there was only a single interview, and for a child to be classified as a picky eater, the caregiver had to respond with a score of at least 3 (“sometimes”) to the question “Is your child a picky eater?”. In the study by Boquin et al. [8], the question “Is your child a picky eater” was asked a total of five times during the research, and to be considered a picky eater, the average score had to be 3 or greater, where scores of 3, 4, or 5 were defined as “sometimes”, “often”, or “always”, respectively. Across the 14 studies, the average age of the children ranged from 4 months to 12.7 years, and the prevalence of picky eating ranged from 6.6 to 59.3%. Of note, when the criteria that defined picky eating were more stringent (e.g., “very picky eater”, “very choosy”, “choosy most of the time”, or “definitely faddy”), the prevalence of picky eating was much lower (e.g., approximately 15% or less).

In the remaining 21 studies, responses to a series of questions were used to determine whether the child was a picky eater [22,23,24,25,26,27,28,29,30,31,32,33,34,35,36,37,38,39,40,41,42]. The tool used most frequently was the Child Eating Behaviour Questionnaire (CEBQ) [43,44], which is a validated 35-item questionnaire, with questions scored on a 5-point Likert scale [responses varied from “never” (score of 1) to “always” (score of 5)]. Six different eating profiles were identified (i.e., “fussy eater”, “moderate eater”, “avoidant eater”, “responsive eater”, “joyful eater”, “approaching eater”), and participants were assigned to one of these profiles based on the highest probability of profile membership. Children classified as having a “fussy eater” profile were characterized by a pattern of high scores in the food avoidance scales (i.e., food fussiness, satiety responsiveness, and slowness in eating) and low scores on the food approach scales (i.e., enjoyment of food and food responsiveness). Within studies, it was demonstrated that how picky eating was defined very much determined the prevalence of picky eating. For instance, in the study by Kwon et al. [22], the prevalence of picky eating was 29.9% when defined by eating a small amount of food and 44.0% when defined by a refusal to eat specific food groups. Likewise, Rydell et al. [42] reported differences in the prevalence of picky eating, depending on where the determination was made: 16% of the children were defined as picky eaters in school but not at home, 6% of the children were defined as picky eaters at home but not in school, while 8.5% of the children exhibited at least one picky eating behavior both at home and in school. Across the 21 studies, the average age of the children ranged from 4 months to 15 years, and the prevalence of picky eating ranged from 5.5 to 70.1%.

As can be seen in Table 1, dietary intakes were assessed in 28 of the studies; across 24 of these studies, the tools used to assess dietary intakes varied and generally included food frequency questionnaires (FFQs) or food records (varying in duration from 1 to 4 days). In the remaining four studies, dietary intakes were assessed experimentally; children were administered standardized test meals, and their acceptability and intakes were assessed by parents [5,8], or the intakes of yoghurts with differing tastes, textures, and colors [28], or pureed versus chopped carrots [36] were assessed. Growth or body weight in picky versus non-picky eaters was assessed in 27 of the studies.

### 3.2. Food/Food Group Intakes in Picky and Non-Picky Eaters

Several researchers have examined whether food intakes, particularly intakes of foods from the major food groups (i.e., cereals/grains, vegetables, fruits, dairy, and meats), differ in children perceived to be picky eaters compared to non-picky eaters, using data collected from dietary intake surveys (i.e., parentally-completed 24-h dietary recalls, food records, or FFQs). These studies are summarized in Table 2 (where the intakes of foods from the different food groups in picky and non-picky eaters are presented), Table 3 (where the intakes of discretionary foods and mixed dishes in picky and non-picky eaters are presented), and Table 4 (where the relationships between picky eater status and the intakes of foods from the different food groups are presented). In addition to the studies summarized in Table 2, Table 3, and Table 4, Boquin et al. [8] noted that non-picky eaters consumed a higher percentage of a standardized meal when compared to picky eaters, while Northstone and Emmett [18] reported that children who were described as “choosy” had lower dietary pattern scores, indicative of their lower variety of foods consumed. Of note, in some of the studies reviewed, the time point at which picky eating behavior was assessed differed from when the food intake data were collected [13,14,19,25,30]. In a few studies, food intakes and picky eating behaviors were both assessed concurrently, as well as at older ages [23,32,37,38].

The intakes of foods from the various food groups were reported differently across the studies. In several of the studies, intakes in picky versus non-picky eaters were reported as amounts of foods consumed (in g/day) from specific food groups [12,14,15] or as the number of servings or cup equivalents of foods from specific food groups [23,39]. In other studies, the results were reported as the odds of consuming foods or a certain number of servings of foods from a specific food group in picky versus non-picky eaters [13,27,35,37,38]. While Carruth et al. [20] reported on the percentage of picky versus non-picky children who consumed foods from the various food groups, Cardona Cano et al. [25] reported on the percentage of picky versus non-picky eaters who did not consume at least 10 g/day of foods from the specific food group. In the remaining studies, differences between picky and non-picky eaters were reported as beta coefficients [13,19,32], dietary intake scores [29,30], or correlation coefficients [40].

Carruth et al. [20] reported that no major differences were evident in the proportion of picky versus non-picky eaters (age 4 to 24 months of age) who consumed foods from each of the major food groups. However, the data were not analyzed statistically, and the intakes of foods from the major food groups were assessed based on a single 24-h dietary recall, with any amount of food that was consumed qualifying the child as a consumer of that food group.

**Fruits and vegetables:** Significantly lower intakes of vegetables were reported in picky versus non-picky eaters in 10 of 13 studies [12,14,19,23,25,30,33,35,39,40]. The intakes of fruits were significantly lower in picky versus non-picky eaters in 7 of the 13 studies [12,14,19,29,32,35,39]. When fruit and vegetable intakes were assessed collectively as one group, intakes were significantly reduced in picky versus non-picky eaters in the majority of analyses conducted by Oliveira et al. [27], but not in the analysis conducted by Dubois et al. [37,38]; however, in this latter study, the odds of consuming five or more servings of fruits and vegetables in picky versus non-picky eaters was assessed.

**Grain and grain products:** There was generally no difference between picky and non-picky eaters in the intakes of grains and grain products [12,13,20,33,37,38,39]; however, when intakes of grains and grain products were separated further into refined, whole grain and starchy grains (e.g., rice, pasta, potatoes), differences between picky and non-picky eaters became more discernible. Specifically, in the study by Cardona Cano et al. [25], intakes of refined grains and grain products were similar between picky and non-picky eaters; but, intakes of whole grains and rice and pasta were significantly lower in picky versus non-picky eaters. Likewise, in the study by Tharner et al. [30], intakes of refined grains and pasta, rice and potatoes, when expressed as z-scores, were not different between picky and non-picky eaters; however, intakes of whole grains, when expressed as z-scores, were significantly lower in picky versus non-picky eaters.

**Dairy intakes:** Dairy intakes—including formula intakes—were largely similar between picky and non-picky eaters [12,20,25,30,33,37,38,39], except in the study by Taylor et al. [14], wherein picky eaters (but not somewhat picky eaters) were noted to have significantly greater intakes of dairy compared to non-picky eaters.

**Meats and meat alternatives:** In the majority of studies, meat intakes were significantly lower in picky versus non-picky eaters [14,25,30,33,37,38]. In two studies, meat intakes between picky and non-picky eaters were not significantly different [20,39], and in one study meat intakes were significantly greater in picky versus non-picky eaters [12]. Intakes of eggs, which were assessed individually only in one study, were significantly lower in picky versus non-picky eaters [12]. Fish intakes were significantly lower in picky versus non-picky eaters in all three studies in which this was assessed [14,25,30]. Intakes of processed meats in picky versus non-picky eaters, which were assessed in a single study, were similar [14].

**Intakes of specific foods or food categories**: In some of the identified studies, the intakes of generally well-liked but unhealthy foods (e.g., desserts, confectionaries, savory snacks, and sugar-sweetened beverages) in picky versus non-picky eaters were assessed. The outcomes of these studies are largely inconsistent. For example, children identified as picky eaters at 1.5 years of age were more likely to refuse confectionaries at the age of 14 months compared to non-picky eaters [25], yet the children who were later identified as fussy eaters at the age of 4 years consumed significantly more quantities of savory snacks and confectionaries when they were younger (at age 14 months) compared to non-fussy eaters [30]. In studies in which eating behaviors and dietary intakes were assessed at the same time point, picky eating was associated with significantly lower intakes of “fats and sweets” in 9-year-old girls [39], but not in two other studies in children 1 to 4 years of age [33] and 2 to 6 years of age [12], in which intakes of sweets and desserts were similar in picky and non-picky eaters. No differences were reported between picky and non-picky eaters in intakes of sugar-sweetened beverages [29,30,32] and savory snacks [33], except in one study in which a significantly higher intake of savory snacks was reported in picky versus non-picky eaters [30]. Overall, there was a limited number of studies in which the intakes of specific foods or categories of foods in picky and non-picky eaters were compared.

### 3.3. Energy, Macronutrient and Fiber Intakes in Picky and Non-Picky Eaters

Several investigators have compared the energy, macronutrient, and fiber intakes of picky eaters and non-picky eaters. The results of these studies are presented in Table 5. It should be noted that in the study by Carruth and Skinner [3], intakes of energy and protein were assessed in picky eaters, but not in non-picky eaters. Thus, intakes of energy and protein in picky eaters were compared to age-specific dietary recommendations by the study authors.

Energy intakes were noted to be significantly lower in picky versus non-picky eaters in six studies [5,16,17,20,22,25,37,38]; but, in nine other studies, energy intakes in picky and non-picky eaters were similar [3,9,12,13,14,17,24,30,39]. The ability to discern a difference in energy intakes between picky and non-picky eaters may be a function of how picky eating is defined. For instance, in the study by Kwon et al. [22], energy intakes were significantly lower in picky eaters versus non-picky eaters when picky eating was defined as “eating small amounts”, but not as “the refusal of ≥2 food groups”. In several of the other studies in which energy intakes were found to be significantly lower in picky versus non-picky eaters, picky eating was defined using more than one qualifying criterion. For example, in the study by Cardona Cano et al. [25], picky eating was defined as “sometimes” or “often” does not eat well and refuses to eat. In the study by Dubois et al. [37,38], picky eating was defined as “always” ate a different meal from that eaten by the family, “often” refused to eat the right food, and “often” refused to eat. In the study by Jacobi et al. [5], caregivers had to answer at least “sometimes” at one interview and “often” at the other interview to the question, “Is your child a picky eater?”.

Intakes of the macronutrients were generally reported in grams/day or as a percentage of total daily energy intake. Protein intake was reported to be significantly lower in picky versus non-picky eaters in five studies [14,16,17,37,38] [only in “very choosy” but not “quite choosy” children]; [13] but not in five other studies [9,12,20,22,39]. Carbohydrate intakes were reported to be significantly lower in picky versus non-picky eaters in two studies [20], in infants aged 7 to 8 months; [16,17]; however, in one study [37,38], the percentage contribution of carbohydrate to total daily energy intake was significantly greater in picky versus non-picky eaters. In the majority of studies, picky and non-picky eaters were found to have similar intakes of carbohydrate [9,12,13,14,17,20,22,39]. Intakes of fat were generally similar in picky and non-picky eaters, with significant differences reported only in three studies. Specifically, fat intakes were reported to be significantly lower in picky versus non-picky eaters in two studies [20], in infants 9 to 11 months but not in infants 7 to 8 months; [37,38] and significantly greater in picky versus non-picky eaters in a single age group in one study [12]. Fiber intakes in picky and non-picky eaters were assessed only in four studies, and although intakes were consistently lower in picky versus non-picky eaters in all four studies, the differences between groups were significant only in two studies [16,39].

The effects of early eating behavior on later intakes of macronutrients were assessed in a limited number of longitudinal studies. Dubois et al. [37,38] reported that children who were considered to be picky eaters at age 2.5, 3.5, or 4.5 years consumed significantly less fat (by 3 g), significantly less protein (by approximately 6 g) and significantly more carbohydrate (by about 1.2 g) at age 4.5 years, in comparison to children who were never reported to be picky eaters. Taylor et al. [14] reported that children with persistent picky eating during the ages of 2.0 to 5.0 years relative to those with no persistent picky eating behaviors had slightly but significantly lower intakes of protein (by approximately 2 g), and slightly but significantly greater free sugar intakes (by approximately 1 g), but similar fat and total carbohydrate intakes at 7.5 years of age.

### 3.4. Micronutrient Intakes in Picky and Non-Picky Eaters

With regard to the intakes of vitamins and minerals (see Table 6, Table 7 and Table 8), Carruth et al. [20] have reported that intakes of certain vitamins and minerals, among infants 7 to 11 months old may be significantly lower among picky eaters in comparison to non-picky eaters. Even so, the study authors noted that the mean levels of intake for all nutrients were well above the recommended dietary allowances (RDA) or adequate intakes (AI) for both picky and non-picky eaters [20]. In a study of 9-year-old girls, picky eaters had significantly lower intakes of vitamin E, vitamin C, and folate [39], and the reduced intakes are likely reflective of their selective food preferences and lower intakes of specific food groups such as fruits and vegetables. Interestingly, the proportion of girls considered to be “at risk” for nutrient inadequacy (i.e., not meeting the Estimated Average Requirements [EAR]) was significantly higher in picky compared to non-picky eaters for vitamin E (98% versus 88%, respectively) and for vitamin C (28% versus 12%, respectively), but not for folate (30% versus 23%, respectively) [39]. Recent studies consistently show lower intakes of iron, zinc, and vitamins A, C, E, B1, B2, and B3 among picky eaters compared to non-picky eaters [12,14,16,17,22], although intakes of most of these nutrients do not appear way below the recommendations. Of note, some nutrients such as iron, zinc, and vitamin D are low among both picky and non-picky eaters. To exemplify this, in the Avon Longitudinal study, one-half to three-fourths of all the children (including both picky and non-picky eaters) had intakes below the recommended levels for iron and zinc, although a significantly higher number of picky eaters had intakes of iron below the recommended intakes when compared to non-picky eaters [14].

### 3.5. Growth/Body Weight Status in Picky and Non-Picky Eaters

The relationship between picky eating and growth has been examined in numerous studies, as summarized in Table 9. It has been reported in some studies that compared to non-picky eaters, picky eaters have statistically significantly lower body mass indices (BMIs) [16,17,22,24,26,30,32,39], lower percentages of body fat [39], and lower fat mass and fat-free mass indices [26]. In some of these studies, when compared to non-picky eaters, picky eaters were also more likely to be classified as being underweight [30], and were less likely to be overweight or obese [24,39]. Picky eaters were reported to have a greater odds of being underweight [34,37]; moreover, having a higher weight-for-age has been associated with significantly lower odds of being a picky eater [20]. In one study, the change in the BMI standard deviation scores between picky eaters and non-picky eaters, which was evaluated from 4 to 6 years, was significantly smaller in picky eaters, mainly driven by a decrease in fat-free mass [26]. Picky eaters have also been reported to be shorter in height compared to non-picky eaters [16,24]. As with the assessment of food intakes, in some of the studies reviewed, the time point at which picky eating behavior was evaluated was not necessarily the same as when growth parameters were measured [13,23,24,26,32,37,38,42].

In contrast, other investigators have not observed any significant associations between anthropometric measures and picky eating behaviors. It was reported in a number of studies that there is no statistically significant difference in mean body weight or height [3,9,34], BMI [1,5,7,28,41], BMI z-scores [13], proportion of children underweight [12,23], overweight [12] or in changes in BMI over a longitudinal follow up of 15 months [13] between picky and non-picky eaters. Rydell et al. [42] reported that “choosy children” were not significantly more likely to have weight:height scores of -1 standard deviation, and van der Horst [33] found that picky eaters were not more likely to be underweight compared to children with less picky eating behaviors. Similarly, Equit et al. [31] reported that “selective eaters” were not significantly more likely to be underweight.

Still, other studies have reported only weak associations between picky eating and growth; for example, Wright et al. [6] reported that children considered to be “faddy” had a slight, non-statistically significant lower body weight, height and Thrive Index (a measure of weight gain starting from birth) compared to children who were not “faddy”. Interestingly, in this same study, children who were perceived to have an “eating problem” did have a statistically significant lower body weight and height and had gained less weight since birth. Chatoor et al. [21] reported that picky eaters had a significantly lower percent ideal body weight compared to non-picky eaters (102.4% versus 107.7%, respectively), but that picky eating status was not a significant predictor of the percent ideal body weight [21].

## 4. Discussion

One of the defining features of picky eating is that the types of foods consumed tend to be limited (i.e., selective eating), with the child exhibiting strong food preferences and food dislikes [1,3,4,6,9]. Parents of picky eaters are also more likely to report that their child does not consume an adequate amount of food at each meal [4,8], or that the child does not eat the amount of food that the parents think they should be eating [3,9].

There is some evidence from the literature to suggest that picky eaters do have less intakes of certain foods/food groups compared to non-picky eaters, when intake was assessed using data collected from dietary intake surveys (24-h dietary recalls, food records, or FFQ). Notably, when compared to non-picky eaters, picky eaters have been reported to consume less fruits and vegetables [12,14,15,19,23,25,27,29,30,32,33,35,39,40], whole grains [25,30], and meat and fish [14,25,30,33,37,38], with the most consistent finding related to the reduced consumption of vegetables in picky versus non-picky eaters. Caution is warranted in interpreting these findings since the extent to which the parents were regulating the child’s food intake is not known. For example, it is unclear whether the lower intake of certain food groups is attributable to the child’s refusal to eat such foods, or whether the foods were simply not offered to the child by the parents, either because the parents know that the child will not eat the food, or for other reasons such as affordability. To avoid these potential confounders, several investigators have evaluated whether food intake differs between picky eaters and non-picky eaters under an experimental setting. Similar to the data collected from dietary intake surveys, the results from two such experimental studies identified in this review suggest that the food choices of picky eaters do differ in some respects when compared to those of non-picky eaters [5,8]. For instance, picky eaters were more likely to avoid vegetables in both of these studies. Thus, vegetable intakes, which tend to be low in general, are even lower in picky eaters.

Varying results have also been reported in studies that included an assessment of energy, macronutrient, and dietary fiber intakes. Although picky eaters had significantly lower intakes of protein than non-picky eaters in some of the identified studies [13,14,16,17,37,38], overall, the intakes of protein were sufficient, both in picky-eaters and non-picky eaters in all age groups assessed. In fact, dietary protein intakes were generally in excess of the European Food Safety Authority (EFSA) dietary recommendations for protein for all age groups. For example, using EFSA’s dietary protein recommendations (specifically, the Population Reference Intakes, which are age- and gender-specific and intended to meet the needs of 97.5% of the population) [45] and reference body weights for European children [46], dietary protein recommendations are 11 to 13 g/day for children 1 to 3 years of age. As per Table 5, dietary protein intakes among children 1 to 3 years of age—irrespective of picky eating behavior—were 39.3 to 50 g/day, representing intakes that were 3.5- to 4.5-fold greater than EFSA’s dietary protein recommendations. On the other hand, fat intakes were generally low in children 1 to 3 years of age (24 to 32% E versus 35 to 40% E of EFSA’s reference intake range [47] for this age group) in both picky eaters and non-picky eaters [3,9,12]; but, in most studies of children 3 years of age and older [16,17,38,39], fat intakes were within EFSA’s reference intake range of 20–35% E for this age group [47]. Dietary fiber intakes need to be increased in children in general, regardless of whether they are a picky eaters or not, although there is evidence to suggest the intake of dietary fiber may be even lower amongst picky eaters [16,39]. The data on consequences of early picky eating behavior [14] and persistent picky eating [38] on later macronutrient intakes are too limited to draw any conclusions. With regard to micronutrients, based on the studies reviewed herein (in at least one of the subgroups examined), picky eaters had significantly lower intakes of certain micronutrients such as iron [14,16,17,20,22], zinc [14,16,17,20], vitamins A [12,16], B6 [12,14,20], C [12,16,20,22], E [16,20,39], thiamine [16,20,22], riboflavin [12,16,20,22], and niacin [14,20,22] compared to non-picky eaters. While intakes in most of these studies were close to recommended values, Taylor et al. [14] noted that the intakes of iron and zinc were below the recommendations in both groups, making it equally important to address these gaps in the general population. Another way to assess nutritional status is to directly measure the levels of micronutrients in blood samples; however, due to the invasive nature of blood sampling, it is not often employed in large-scale studies of young children. Two recent studies have measured the nutrient status of pre-school- and school-aged children and reported significantly lower levels of iron, magnesium, and copper in the blood of school-aged picky eaters compared to non-picky eaters [16], although no such differences were observed in preschool children [17]. If the school-aged picky eaters were picky eaters earlier in life, then there is some suggestion that the persistence of picky eating may have adverse consequences on nutritional status in the long-term.

Rather than examining food/nutrient intakes individually, perhaps a better approach would be to use dietary indices and/or dietary patterns reflecting the overall quality of the diet or adherence to a dietary recommendation, the results of which may be easily understood by the general population. As an example, the Youth Healthy Eating Index (HEI) [48] was constructed for use specifically in children and adolescents. Other examples are the Finnish Children Healthy Eating Index (FCHEI) [49] and the Chinese Children Dietary Index [50]; both scoring systems were developed to assess overall diet quality among children in their respective countries.

There does not appear to be a clear association between picky eating and childhood growth/body weight status across the studies identified in this current review. Similarly, in a recent systematic review, Brown and colleagues [51] reported inconsistent findings among the studies evaluating whether associations exist between picky eating or food neophobia and weight status; in comparison with picky eating or food neophobia, 17 studies found no association with weight status, 2 studies found a positive association with overweight status, 5 studies found a negative association with overweight or obesity status, 6 studies found a positive association with underweight status, and 11 studies found a decreased association with BMI or BMI z-score. It should be noted, however, that studies that used a larger number of qualifying criteria in their classification of picky eating generally reported a significant difference in the growth/weight status of picky versus non-picky eaters. Among the seven studies [16,20,26,30,32,37,38] in which all parameters related to growth/weight were significantly poorer in picky versus non-picky eaters, only in two studies [16,20] was the picky eating classification based on a single question; in the remaining five studies [26,30,32,37,38], the classification of the children as picky eaters was based on multiple defining criteria. Likewise, among the seven studies [6,17,21,22,23,24,34] in which some but not all parameters related to growth/body weight were significantly inferior in picky versus non-picky eaters, only in three studies [6,17,21] was the picky eating classification based on a single question, while in the remaining four studies [22,23,24,34], the classification of the children as picky eaters was based on multiple defining criteria. In the 12 studies [1,3,5,7,9,12,13,28,31,33,41,42] in which there was no difference between picky and non-picky eaters in growth/body weight, in the majority of studies (i.e., seven), the classification of picky eating was based only on a single question. Perhaps a single question is not sufficient to differentiate picky from non-picky eaters, and the greater the number of defining criteria, the more accurately true picky eating can be identified. In addition to how picky eating was defined, there are other factors that could have contributed to the lack of a consistent finding across the studies (e.g., whether the growth parameters were parentally reported or were assessed directly by investigators and the actual growth parameter assessed). Moreover, the studies reviewed herein consisted mainly of healthy children without any formal diagnosis of eating disorders.

The lack of consistent findings between picky eating and food/nutrient intake or growth/body weight status across the different studies in the literature highlights the various challenges faced by researchers in this field. For one, there is no concise definition for picky eating that has been widely adopted in the literature. There are several different ways by which researchers have identified picky eaters or defined picky eating in studies, ranging from the simple question, “Is your child a picky eater?”, which is highly subjective and requires interpretation by the responder, to the use of more complex tools that rely on a combination of responses related to picky eating behaviors. A single question may not be sufficient to delineate between parental perception of picky eating and true picky eating, and so a tool that incorporates several different eating behaviors that are problematic in picky eaters, such as the CEBQ, may be more sensitive in identifying true picky eating. Understandably, there are many difficulties in deriving a widely accepted definition of what constitutes picky eating. As with any other human behavior, picky eating is highly complex; it is constantly evolving even within an individual and is likely to change with age. However, many of the studies identified herein were cross-sectional in design. Therefore, more longitudinal studies are needed to better characterize picky eating over time, to better understand whether such behaviors are transient phenomena or persist over time, and to identify the most relevant and sensitive age range wherein picky eating has the greatest impact on food choices or health outcomes later in life. Finally, some consensus on the most sensitive or pertinent dietary intake and growth measures is needed, as the results for these outcomes were reported with heterogeneity across the studies. The tools used to assess dietary intakes varied across the studies, with some using a single 24-h dietary recall, others using multiple 24-h recalls, and others a FFQ. The way the results were expressed was also highly variable. For instance, across studies, the intake of fruits and vegetables in picky and non-picky eaters was expressed as amount (g) per day, the proportion of children consuming a minimum amount, or as an odds ratio. Likewise, for body weight, there was little consistency in how the outcomes were reported across studies (e.g., z-score, BMI, OR of being underweight, normal weight, or over weight, etc.). 

There is a wealth of literature published on picky eating during childhood, and across a broad range of ages, as evidenced from this review. There appears to be the general consensus that picky eating (or “selective food choice”), to some extent, is a normal part of the child development process and does not negatively impact growth or nutritional status. However, on an individual basis, it is important to differentiate picky eating behaviors from the more serious eating disorders that could have negative implications on health (e.g., growth impediment, nutritional deficiency, or other functional impairments). Efforts should be made to ensure that all children, especially those with perceived picky eating, consume a nutritionally balanced and varied diet in accordance with the recommendations set forth in the current dietary guidelines. It is important to continue to promote healthy eating habits among children in general, particularly in children with perceived picky eating, by providing repeated exposure to a variety of foods, offering age-appropriate textures/portion sizes, using appropriate feeding techniques, practicing responsive feeding, and role modelling of food choices.

The limitations of this narrative review should be mentioned. First, the evidence is predominantly from cross-sectional studies, and it is well-known that dietary recall can be biased in such studies (e.g., parents of children who perceive their children as picky eaters may indicate poorer dietary intakes/habits than what the child actually exhibits). A limited number of longitudinal studies were identified, and such studies are important in understanding whether picky eating is a transient phenomenon with no long-term effects on growth or nutritional status or if picky eating is sustained, with more detrimental effects on nutritional status and growth in the long-term. Another limitation of this review is that the assessment presented herein is strictly qualitative—we did not pool results across studies (for example, the intakes of fruits and vegetables in picky versus non-picky eaters), and it is possible that a quantitative assessment (such as that afforded by a meta-analysis) may have increased our sensitivity in identifying differences between picky and non-picky eaters; however, the studies identified are too heterogeneous to pool (i.e., the studies differed considerably in how picky eating was defined, how dietary intakes were assessed, and how growth was monitored). Finally, given that the interest was in several different aspects of picky eating, such as diagnostic criteria, dietary intakes, body weight/growth status, and overall nutritional adequacy in picky versus non-picky eaters, the research was conducted and reported as a narrative review as opposed to a systematic review, and so it is likely that not all relevant studies have been captured. Nonetheless, the sampling of studies presented herein is robust, and the heterogeneity in critical research elements is evident. Research in the area of picky eating would benefit from increased alignment in how clinical studies are designed, how picky eating is identified, and the best methods for assessing and reporting nutritional intakes and growth. 

## Figures and Tables

**Table 1:** Key Characteristics of the Identified Studies ^a^.

Reference	Study Design, Country	Sample Size, Age	Classification of Picky Eating	Prevalence of Picky Eating ^b^	Assessment of Growth	Method of Dietary Intakes Assessment	Growth and/or Dietary Intakes Assessed Concurrently with Picky Eating
**Use of a Single Closed-ended (Yes/No) Question (*n* = 3 studies)**							
Li et al., 2017 [12]	Cross-sectional PRC	*n* = 1414 6 to 35 mo	Caregiver responded “**yes**” when asked if their child was a PE.	12.3% to 36.1%	Yes	One 24-h recall.	Yes
Carruth and Skinner, 2000 [3]	Longitudinal U.S.	*n* = 71 34 to 84 mo	Caregiver responded “**yes**” when asked if their child was a PE.	*30%* to *49%*	Yes	2-day food record and one 24-h dietary recall.	Yes
Carruth et al., 1998 [9]	Cross-sectional U.S.	*n* = 118 24 to 36 mo	Caregiver responded “**yes**” when asked if their child was a PE.	36%	Yes	2-day food record and one 24-h dietary recall.	Yes
**Use of a Single Question with a Response Selected from Several Possible Responses [*n* = 14 studies]**							
Rohde et al., 2017 [13]	Cross-sectional Denmark	*n* = 271 2 to 6 y	Parent responded “**picky**” or “**a little picky**” to: “How would you describe your child’s way of eating?”	**Picky:** 16% **A little picky:** 42%	Yes	4-day dietary records.	No. Growth and food intakes were assessed 15 mo after the assessment of picky eating.
Taylor et al., 2016 [14]	Longitudinal UK	*n* = 7420 2 to 7.5 y	Caregiver responded “**no**”, “**yes, quite choosy**”, or “**yes, very choosy**” to: “Does your child have definite likes and dislikes as far as food is concerned?”	9.7% to 14.7%	No	3-day food record.	No. picky eating was assessed when children were aged 2, 3, 4.5, and 5.5 y of age. Dietary intakes were assessed at 3.5 and 7.5 y of age.
van der Horst et al., 2017 [15]	Cross-sectional U.S.	*n* = 2371 1 to 4 y	Caregivers responded either “**a very picky eater**”, or “**a somewhat picky eater**” to: “Is your child a PE?”	**Somewhat PE:** 27.9% to 40.4% **Very PE:** 6.6% to 15.2%	No	One 24-h food recall.	Yes
Xue et al., 2015 [16]	Cross-sectional PRC	*n* = 793 7 to 12 y	Caregiver responded “**sometimes” or “always**” to: “Is your child a PE?”	59.3%	Yes	24-h dietary record/recall on weekdays.	Yes
Xue et al., 2015 [17]	Cross-sectional PRC	*n* = 937 3 to 7 y	Caregiver responded “**sometimes” or “always**” to: “Is your child a PE?”	54%	Yes	24-h dietary record on weekdays and FFQ.	Yes
Boquin et al., 2014 [8]	Cross-sectional study and in-home meal test study U.S.	*n* = 170 2 to 4 y	Child was considered a PE if the average score was ≥3 to the question: “Is your child a picky eater?”; possible responses were 1=never, 2=rarely; 3=sometimes; 4=often; 5=always.	*48.8%*	No	Over a 2-week in-home meal study, parents rated their own and their child’s liking of standardized meals.	Yes
Northstone and Emmett, 2013 [18]	Cross-sectional UK	*n* = 9599 2 y	Caregiver responded “**yes, quite choosy**” or “**yes, very choosy**” to the statement: “Child has definite likes/dislikes”.	**Yes, quite choosy:** 31.2% **Yes, very choosy:** 9.8%	No	FFQ.	Yes
Jones et al., 2010 [19]	Cross-sectional UK	*n* = 7285 7 y	Caregiver responded “**yes most of the time**” or “**sometimes**” to the statement: “He/she is choosy about food”.	**Choosy most of the time:** 15.8% **Choosy sometimes:** 40.8%	No	Three 24-h unweighted food records.	No. picky eating behavior was assessed at 65 mo of age. Dietary intakes were assessed at 7 y of age.
Mascola et al., 2010 [7]	Cross-sectional U.S.	*n* = 120 11 y	Caregivers responded either “**often**” or “**always**” to: “Is your child a PE?”	22%	Yes	Not assessed.	Yes
Jacobi et al., 2008 [1]	Cross-sectional Germany	*n* = 426 7.7 to 12.7 y	Caregivers responded with at least “**sometimes**” to: “Is your child a PE?”	*19%*	Yes	Not assessed.	Yes
Wright et al., 2007 [6]	Cross-sectional UK	*n* = 455 30 mo	Caregiver responded “**definitely**” to the question regarding their child’s faddy eating behavior.	**Definitely faddy:** 8% **Maybe faddy:** 15% **Eating problem:** 20%	Yes	Not assessed.	Yes. The change in weight starting from birth was used to calculate the Thrive Index.
Carruth et al., 2004 [20]	Cross-sectional U.S.	*n* = 2854 4 to 24 mo	Caregivers responded that their child was a “**very PE**” or a “**somewhat PE**”.	19% to 50%	Yes	One 24-h dietary recall.	Yes
Jacobi et al., 2003 [5]	Cross-sectional U.S.	*n* = 135 3.5 and 5.5 y	Caregivers responded with at least “**sometimes**” (score of 3) to: “Is your child a PE?” at one of the two interviews and at least “**often**” (score of 4) at the other interview.	21%	Yes	One laboratory and two 24-h in-home standardized food intake tests.	Yes
Chatoor et al., 2000 [21]	Case-control U.S.	*n* = 68 12 to 37 mo	Caregiver described their child as being “**often**” or “**always**” a PE.	Not applicable	Yes	Not assessed.	Yes
**Use of Several Questions and a Combination of Responses [*n* = 21]**							
Kwon et al., 2017 [22]	Cross-sectional Korea	*n* = 184 1 to 5 y	Caregivers responded using a five-point scale of 1 (almost never) to 5 (almost always) to four specific questions relating to picky eating.	Overall prevalence: 70.1% Eating small amount: 29.9% Refusal to eat specific food groups: 44.0%	Yes	Non-consecutive 3-day diet records.	Yes
Berger et al., 2016 [23]	Longitudinal U.S.	*n* = 181 5 to 15 y	Three items from the Pickiness Subscale of the CFQ were each scored out of five. A mean score for PE was calculated at each occasion. Persistent picky eating from ages 5 to 9 y was defined as having a mean PE score >3 at ≥2 of 3 timepoints.	Persistent PE from ages 5 to 15 y: 18%	Yes	Three 24-h dietary recalls at each occasion.	Somewhat. Picky eating was assessed at 5, 7, and 9 y of age. Dietary intakes and growth were assessed at 5, 7, 9, 11, 13, and 15 y of age.
Antoniou et al., 2016 [24]	Longitudinal The Netherlands	*n* = 1024 5 to 9 y	Mothers responded on a 5-point Likert scale ranging from “strongly agree” to “strongly disagree” with the following statements: “My child’s diet consists of only few foods”, “My child is unwilling to eat many of the foods I serve”, and “My child is picky or fussy about what s/he eats”.	5 y: 39.3%	Yes	FFQ (at 5 y of age).	No. Picky eating behaviors and food parenting practices were assessed at 5 y of age. Growth was assessed at 5, 7, 8, and 9 y of age.
Cardona Cano et al., 2015 [25]	Longitudinal The Netherlands	*n* = 3618 to 4018 1.5 to 6 y	Caregiver response of “**sometimes**” and/or “**often**” on two items of the CBCL that were used to assess picky eating: “does not eat well” and “refuses to eat”.	13.2% to 27.6%	No	FFQ of foods consumed over the past 4 weeks.	No. Eating behaviors were assessed at 1.5 years of age, while dietary intakes were assessed at 14 mo of age.
de Barse et al., 2015 [26]	Longitudinal The Netherlands	*n* = 4,191 4 to 6 y	Latent profile analysis of responses to the CEBQ to come up with a “fussy eater” profile.	4 y: 5.7%	Yes	Not assessed.	No. Eating behaviors were assessed at 4 y of age. Height, weight, and body composition were assessed at 6 y of age.
Oliveira et al., 2015 [27]	Longitudinal analysis of 3 cohorts: G2I (Portugal), ALSPAC (UK), and EDEN (France)	G 2I (*n* = 4227), ALSPAC (*n* = 7620) EDEN (*n* = 892) 0.3 to 5 y	Caregiver’s perception (based on yes/no questions) of feeding difficulties	**% Difficulties in Eating:** 21 to 66% **% Food refusal:** 50 to 66%	Yes, but not in relation to PE status	FFQ at age 4 to 5 y.	Yes, only at 4 to 5 y of age.
Werthmann et al., 2015 [28]	Experimental study The Netherlands	*n* = 32 2.5 to 4 y	Child’s fussiness was assessed by parental responses to the food fussiness sub-scale of the CEBQ.	Not reported	Yes	Intakes of yoghurts after manipulating taste, texture, and color were assessed.	Yes
Haszard et al., 2010 [29]	Cross-sectional New Zealand	*n* = 203 4 to 8 y	Fussy eating was assessed using the average of four questions from the Lifestyle and Behaviour Checklist.	36.5%	No	Assessed using three scales from the CDQ.	Yes
Tharner et al., 2014 [30]	Cross-sectional The Netherlands	*n* = 4915 4 y	Latent profile analysis of responses to the CEBQ to come up with a “fussy eater” profile.	5.6%	Yes	FFQ of foods consumed over the past 4 weeks.	No. Eating behaviors, weight, and height were assessed at 4 y of age, while dietary intakes were assessed at 14 mo of age.
Equit et al., 2013 [31]	Cross-sectional Germany	*n* = 1090 4 to 7 y	Latent class analysis to identify three distinct groups of children with different patterns of eating behaviors (Class 1: “normal eaters”; Class 2: “weight worriers”; Class 3: “selective eaters”).	34.1% were identified as Class 3: “selective eaters”	Yes	Not assessed.	Yes
Rodenburg et al., 2012 [32]	Longitudinal and cross-sectional The Netherlands	*n* = 1275 7 to 10 y	Child’s fussiness was assessed by parental responses to the food fussiness sub-scale of the CEBQ.	Not reported	Yes	Assessed using a questionnaire that was based on a validated FFQ.	No. The CEBQ was completed in 2009, and weight and food intakes were assessed in 2009 and again in 2010.
van der Horst, 2012 [33]	Cross-sectional Switzerland	*n* = 305 6 to 12 y	Caregivers responded with a pattern of high scores on the CEBQ.	*45%*	Yes	Not assessed.	Yes
Ekstein et al., 2010 [34]	Case-control Jerusalem	*n* = 170 14 to 91 mo	Unwillingness to eat familiar foods or try new foods, severe enough to interfere with daily routines to an extent that was problematic to the parent, child, or parent-child relationship.	Not applicable	Yes	Not assessed.	Yes
Horodynski et al., 2010 [35]	Cross-sectional U.S.	*n* = 399 25 mo (mean)	The five-item PE sub-scale of the TPMBQ was used.	Not reported	Yes, but not in relation to PE status	A FFQ, adapted from the Block FFQ, was used.	Yes
Blossfeld et al., 2007 [36]	Experimental study Ireland	*n* = 70 48 to 57 weeks	“**Pickiness**” was assessed using answers to six questions scored on a 7-point Likert scale. “**Fussiness**” was assessed using the Fussiness sub-scale of the CEBQ.	Not reported	Yes, but not in relation to PE status	Intakes of pureed versus chopped carrots were assessed.	Yes
Dubois et al., 2007 [37,38]	Longitudinal Canada	*n* = 1498 2.5 to 4.5 y	Caregiver responded that he/she: “**always** ate a different meal from that eaten by the family”; “**often** refused to eat the right food”; and “**often** refused to eat”.	Defined as PE at all 3 timepoints: 5.5%	Yes	One 24-h food recall.	No. Eating behaviors were assessed when the child was 2.5, 3.5, and 4.5 y old. Dietary intakes, weight, and height were assessed only at age 4.5 y.
Galloway et al., 2005 and 2007 [39,40]	Cross-sectional U.S.	*n* = 173 to 189 7 y, 9 y	Three items from the Pickiness Subscale of the CFQ were each scored out of five. Picky eating was categorized using the median split of the total score, which was derived as the mean score of the three items.	47% (7y) 48% (9 y)	Yes (at 9 y only)	Three 24-h dietary recalls over a 2-week period.	Yes
Lewinsohn et al., 2005 [41]	Cross-sectional U.S.	*n* = 93 36 mo	Exploratory factor analysis on caregiver responses to the ORI-CEBI.	Not reported	Yes	Not assessed.	Yes
Rydell et al., 1995 [42]	Cross-sectional Sweden	*n* = 240 6.1 to 11.0 y	Caregiver or teacher indicated at least two of the behaviors (eats small portions, refuses foods, disinterested in food/does not appear hungry), with behaviors occurring at least three to four times per week.	6 to 16%	Yes	Not assessed.	No. It appears that weight and height were assessed at different time points from the child’s eating behaviors. The difference in time points is not clearly stated, but the wording in the publication suggests this may be 1 to 2 y.

Abbreviations: ALSPAC = Avon Longitudinal Study of Parents and Children; CBCL = Child Behavior Checklist; CDQ = Children’s Dietary Questionnaire; CEBQ = Children’s Eating Behaviour Questionnaire; CFQ = Child Feeding Questionnaire; EDEN = Study on the pre- and early postnatal determinants of child health and development; FFQ = food frequency questionnaire; G21 = Generation XXI; h = hour; mo = months; n = number; ORI-CEBI = Oregon Research Institute Child Eating Behavior Inventory; PE = picky eater(s); PRC = People’s Republic of China; TPMBQ = Toddler–Parent Mealtime Behavior Questionnaire; UK = United Kingdom; U.S. = United States; y = years. ^a^ Values in italics were calculated using information provided in the publication. ^b^ If the prevalence of picky eating was reported for individual age groups in the publication, they are summarized as a range in this table.

**Table 2:** Food Preferences Assessed as Intakes of Foods from Major Food Groups in PE and NPE ^a^.

Reference	Measure, Age at Intake Analysis	Fruits		Vegetables		Grains and Grain Products		Dairy		Meats and Meat Alternatives	
		PE	NPE	PE	NPE	PE	NPE	PE	NPE	PE	NPE
Li et al. [12]	Intake as g/d, reported as mean ± SD 6 to 35 mo	45.3 ± 78.9 *	**78.9 ± 121.8**	66.2 ± 87.6	52.8 ± 72.8	117.3 ± 82.2	154.1 ± 139.3	298.1 ± 214.9	223.6 ± 208.9	**Meat**	
										67.5 ± 80.2 *	**49.1 ± 88.8**
										**Eggs**	
										**26.1 ± 43.8**	**36.9 ± 44.4**
van der Horst et al. [15]	Intake (g/d), reported as mean ± SEM 1 to 4 y	254 ± 12	263 ± 9	57.9 ± 3.8 *	**69.7 ± 4.1**	101 ± 4	100 ± 4	470 ± 15	458 ± 13	60.9 ± 3.2 *	**76.9 ± 3.9**
Taylor et al. [14]	Intake (g/d), reported as mean (95% CI) 3.5 y	**46** (36, 56) ^b,^*	**72** **(65, 78)**	**25** (19, 31) ^b,^*	**52** **(48, 57)**	--		390 (342, 437) ^b^*	**325 (305, 344)**	**Total meat ^b^**	
										50 (43, 57) *	**61 (57, 65)**
										**Processed meat ^b^**	
										24 (20, 28)	23 (20, 25)
										**Fish ^b^**	
										9 (7, 12) *	**16 (14, 18)**
		68 (62, 75) ^c^	72 (65, 78)	42 (38, 46) ^c^	52 (48, 57)	--		347 (322, 372) ^c^	325 (305, 344)	**Total meat ^c^**	
										52 (48, 56) *	**61 (57, 65)**
										**Processed meat ^c^**	
										23 (21, 26)	23 (20, 25)
										**Fish ^c^**	
										15 (13, 17)	16 (14, 18)
Berger et al. [23]	Intake in PPE and NPE, reported as mean ± SEM of cup equiv ^d^ 5 to 15 y	0.70 ± 0.10	0.72 ± 0.05	0.57 ± 0.06 *	**0.73 ± 0.03**	--		--		--	
Cardona Cano et al. [25]	% of children not eating ≥10 g of food from the specific food group 14 mo	4.9	3.8	46.7 *	**36.0**	**Refined**		**Dairy**		**Fish**	
						58.8	58.9	32.5	29.3	89.1 *	**86.0**
						**Whole**		**Formula**		**Meat**	
						15.8 *	**10.6**	29.2	31.4	64.8 *	**58.7**
						**Rice, pasta**					
						22.8 *	**16.6**				
Tharner et al. [30]	Intake, z-score 14 mo	0.03	0.04	−0.21 *	**−0.01**	**Refined**		**Dairy**		**Fish**	
						0.05	−0.50	−0.13	0.01	−0.16*	**0.0**
						**Whole**		**Formula**		**Meat**	
						−0.20 *	**0.08**	0.07	−0.01	−0.18 *	**0.04**
						**Pasta, rice, potatoes**					
						−0.16	−0.02				
Haszard et al. [29]	Dietary intake scores mean ± SD 4 to 8 y (mean 6.4 y)	**Rec. score ≥ 1**				--		--		--	
		12.3 ± 3.9 *		**14.5 ± 4**							
Carruth et al. [20] ^e^	% of children eating food from category 4 to 6 mo	64	57	50	47	79	77	100	100	7	5
	7 to 8 mo	83	90	67	68	88	92	100	100	20	13
	9 to 11 mo	89	89	65	76	96	96	100	100	33	45
	12 to 14 mo	84	94	72	79	98	98	100	99	72	75
	15 to 24 mo	87	84	77	84	95	98	97	97	86	89
Galloway et al. [39]	# of servings per d, reported as mean ± SD 9 y	1.0 ± 0.94 *	**1.5 ± 1.1**	**1.3 ± 0.72**	**1.7 ± 0.89**	5.9 ± 1.6	6.3 ± 1.8	2.9 ± 1.3	2.8 ± 1.2	1.4 ± 0.59	1.5 ± 0.63

Abbreviations: CI = confidence interval; d = day; mo = months; NPE = non-picky eaters; PE = picky eaters; PPE = persistent picky eaters; Rec. = recommended; SD = standard deviation; SEM = standard error of the mean; y = years. ^a^ Values appearing in bold-type font and with an asterisk (*) are significantly different between PE and NPE. ^b^ Results for those with score 2 (PE). ^c^ Results for those with score 1 (somewhat PE). ^d^ Recommend intakes of cup equiv: fruit = 3/d and vegetables = 2/d. ^e^ Significance between PE and NPE within each age group was not reported.

**Table 3:** Food Preferences Assessed as Intakes of Discretionary Foods and Mixed Dishes in PE and NPE ^a^.

Reference	Measure, Age at Intake Analysis	Desserts		Sugar-sweetened Beverages			Savoury Snacks		Mixed Dishes	
		PE	NPE	PE		NPE	PE	NPE	PE	NPE
Li et al. [12]	Intake as g/d, reported as mean ± SD 6 to 35 mo	409 ± 362	439 ± 351	--		--	--	--	--	--
van der Horst et al. [15]	Intake (g/d), reported as mean ± SEM 1 to 4 y	161 ± 10 ^b^	140 ± 8 ^b^	--		--	140.5 ± 7.8	161.4 ± 10.1	95 ± 6	110 ± 6
Cardona Cano et al. [25]	% of children not eating ≥10 g of food from the specific food group 14 mo	**Confections**		--			87.1	87.0	24.1	21.9
		6.2*	**4.1**							
Tharner et al. [30]	Intake, z-score 14 mo	**Confections**		−0.05	−0.01		0.16 *	**0.06**	**RTE**	
		0.15 *	**−0.04**							
									0.22	0.01
Haszard et al. [29]	Dietary intake scores mean ± SD 4 to 8 y (mean 6.4 y)	--		**Rec. score ≤ 1**			**Rec. score ≤ 2**		--	
				0.9 ± 1.1	1.0 ± 1.4		2.6 ± 0.9	2.4 ± 1.0		
Carruth et al. [20] ^c^	% of children eating food from category 4 to 6 mo	14 ^b^	12 ^b^	^--^			41	41	14	13
	7 to 8 mo	48 ^b^	45 ^b^	--			64	59	52	46
	9 to 11 mo	58 ^b^	61 ^b^	--			69	75	58	66
	12 to 14 mo	77 ^b^	76 ^b^	--			86	87	68	72
	15 to 24 mo	86 ^b^	89 ^b^	--			93	93	67	71
Galloway et al. [39]	# of servings per d, reported as mean ± SD 9 y	4.9 ± 2.1 *	**5.8 ± 2.8**	--			--		--	

Abbreviations: d = day; mo = months; NPE = non-picky eaters; PE = picky eaters; Rec. = recommended; RTE = ready to eat; SD = standard deviation; SEM = standard error of the mean; y = years. ^a^ Values appearing in bold-type font and with an asterisk (*) are significantly different between PE and NPE. ^b^ Includes sweets, sweetened beverages and dessert. ^c^ Significance between PE and NPE within each age group was not reported.

**Table 4:** Food Preferences Assessed as a Function of PE Status ^a^.

Reference	Relationship Measure	Age at Analysis		Food/Food Group Assessed	Results
Rohde et al. [13]	β (95% CI) (relative to NPE) ^b^	2 to 6 y (median 3.9 y)		Fruits	2.9 (−18.1 to 24.0)
				Vegetables	0.24 (−24.55 to 25.02)
				Grains and Grain Products	−10.16 (−23.25 to 2.93)
Oliveira et al. [27] ^c^	OR (95% CI) for relation-ship between early eating behavior and later high fruit and vegetable intakes ^d^.	Feeding difficulties	4 to 6 mo	Fruits/Vegetables	G21, 0.74 (0.5, 1.03) ALSPAC, 0.82 (0.72, 0.93) *
			12 to 15 mo		G21, 0.69 (0.48, 0.99) * ALSPAC, 0.77 (0.67, 0.88) *
			24 mo		ALSPAC, 0.76 (0.67,0.86) * EDEN, 0.67 (0.49,0.93) *
			48 to 60 mo		G21, 0.66 (0.58, 0.76) * ALSPAC, 0.75 (0.67,0.84) * ****EDEN, 0.72 (0.51,1.02)
		Poor eating	4 to 6 mo		G21, 1.13 (0.82, 1.56) ALSPAC, 1.07 (0.94,1.22) EDEN, 1.40 (0.67,2.93)
			12 to 15 mo		G21, 0.63 (0.44, 0.92) * ****ALSPAC, 1.03 (0.9,1.17) EDEN, 0.82 (0.44,1.48)
			24 mo		ALSPAC, 0.87 (0.76, 0.99) * ****EDEN, 1.12 (0.63,1.98)
			48 to 60 mo		G21, 0.71 (0.62, 0.81) * ALSPAC, 0.80 (0.71,0.91) *
		FR/food neophobia	4 to 6 mo		G21, 0.87 (0.64, 1.19) ALSPAC, 1.07 (0.92,1.26)
			12 to 15 mo		G21, 0.72 (0.50, 1.04) ALSPAC, 0.89 (0.78,1.01)
			24 mo		ALSPAC, 0.84 (0.72, 0.98) * EDEN, 0.60 (0.46,0.79) *
			48 to 60 mo		G21, 0.58 (0.51, 0.67) * ALSPAC, 0.85 (0.76,0.96) * EDEN, 0.62 (0.47,0.82) *
Rodenburg et al. [32]	Adjusted β^q^ (relationship between food fussiness and food intake)	7 to 10 y (2009 analysis)		Fruits	β = −0.16 *
				Sugar/Sweetened Beverages	β = 0.03
		8 to 11 y (2010 analysis)		Fruits	β = -0.14 *
				Sugar/Sweetened Beverages	β = 0.04
Horodynski et al. [35]	Odds of consumption for PE vs. NPE	25 mo		Fruits	OR = 0.641; 95% CI: 0.452 to 0.908 *
				Vegetables	OR = 0.397; 95% CI: 0.271 to 0.582 *
Jones et al. [19]	β is the adjusted difference in intake relative to the reference category “choosy most of the time”, which is set at zero.	7 y		Fruits	Choosy sometimes: β = 1.82; 95% CI: 0.67 to 2.97 Not choosy: β = 2.77; 95% CI: 1.62 to 3.91 * P for trend: <0.001
				Vegetables	Choosy sometimes: β = 1.64; 95% CI: 1.03 to 2.24 * Not choosy: β = 2.57; 95% CI: 1.87 to 3.27 * **P for trend: <0.001**
Dubois et al. [37,38]	Odds of eating specified # of servings from each food group relative to the group for which picky eating was “never reported”, which was set at 1.0.	4.5 y ^e^		Fruits/Vegetables (>5 servings)	PE reported once or twice: OR = 0.733; 95% CI: 0.508 to 1.058 PE reported at all 3 ages: OR = 0.570; 95% CI: 0.278 to 1.169
				Grain products (>5 servings)	PE reported once or twice: OR = 0.781; 95% CI: 0.545 to 1.118 PE reported at all 3 ages: OR = 0.635; 95% CI: 0.317 to 1.272
				Milk products (≥2 servings)	PE reported once or twice: OR = 0.831; 95% CI: 0.646 to 1.069 PE reported at all 3 ages: OR = 0.843; 95% CI: 0.532 to 1.335
				Meat and alternatives (≥2 servings)	PE reported once or twice: OR = 0.628; 95% CI: 0.479 to 0.823 * PE reported at all 3 ages: OR = 0.319; 95% CI: 0.181 to 0.560 *
Galloway et al. [40]	r_s_ (value for relationship between pickiness and food intake level).	7 y		Vegetables	r_s_ = −0.21 *

Abbreviations: ALSPAC = Avon Longitudinal Study of Parents and Children; β = regression coefficient; CI = confidence intervals; EDEN = Study on the pre- and early postnatal determinants of child health and development; FR = food refusal; G21 = Generation XXI; mo = months; NPE = non-picky eater(s); OR = odds ratio; PE = picky eater(s); r_s_ = Spearman correlation coefficient; y = years. ^a^ Values appearing in bold-type font and with an asterisk (*) are significantly different between PE and NPE. ^b^ Values reported (β coefficients and 95% CI) are the results of linear regression models [adjusted for baseline measures of outcome, group (intervention/control), age, sex, and maternal education]. ^c^ “Feeding difficulties” corresponds to feeding difficulties (ALSPAC, GEN21, EDEN), “poor eating” corresponds to eats small quantities at each meal (at 4 to 5 mo) and does not eat enough (at 12 to 15 mo) (ALSPAC; Generation 21) and needs to be stimulated (at 4, 12 and 24 mo)(EDEN). “Food refusal” corresponds to refusal of milk (at 4 to 6 mo) and solids (at 12 to 15 mo) (ALSPAC, G21). “Food neophobia” was calculated as the mean of three questions [≥median (more neophobic) vs. <median (reference category)] (EDEN). ^d^ A high fruit intake was defined as >1 serving/d, and a high vegetable intake was defined as >1 serving/d, except in G21 (>3 serving/d). Subsequent intakes were assessed at age 4 y in G21, 4.5 y in ALSPAC, and 5 y in EDEN. ^e^ Eating behavior was assessed at 2.5, 3.5, and 4.5 years. A NPE was defined as a child for whom picky eating was not reported at any of the three ages. A PE was defined in two ways: (i) as a child for whom picky eating was defined at one or two ages (“reported once or twice”); or (ii) a child for whom picky eating was defined at all three ages (reported at all three ages).

**Table 5:** Intakes of Energy and Macronutrients in PE and NPE ^a^.

**Reference**	**Age Range at Intake Analysis** **** **(mean)**	**Energy** **[kcal/day (kJ/day), (kJ/kg BW)]**		**Carbohydrate** **[g/d (%E)]**		**Protein** **[g/d (%E)]**		**Fat** **[g/d (%E)]**		**Fiber** **(g/d)**	
		**PE**	**NPE**	**PE**	**NPE**	**PE**	**NPE**	**PE**	**NPE**	**PE**	**NPE**
Rohde et al. [13] ^b^	2 to 6 y (3.5 y)	−121.3 (−487.8; 245.1)	--	0.72 (−1.20; 2.63)	--	**−1.17** (−2.02; −0.32) *	--	0.29 (−1.50; 2.09)	--	--	--
Kwon et al. [22] ^c^	1 to 5 y (2.8 y)	1155 ^d,^*	**1340**	*173.3* (60%) ^d^	*204.4* (61%)	*46.2* (16%) ^d^	*53.6* (16%)	*30.8* (24%) ^d^	*35.7* (24%)	--	--
		1261 ^e^	1304	*189.2* (60%) ^e^	*204.4* (61%)	*50.4* (16%) ^e^	*53.6* (16%)	*33.6* (24%) ^e^	*35.7* (24%)	--	--
Li et al. [12]	6 to 11 mo	727	744	108.3 (59.6*%*)	105 (*56.5%*)	23.5 (*12.9%*)	24.2 (*13.0%)*	23.2 (*28.7%*)	25.7 (*31.1%*)	1.7 (*0.47%*)	2.1 (*0.56%*)
	12 to 23 mo	1108	1146	150.1 (*54.2%*)	154.8 (*54.0%*)	39.3 (*14.2%*)	41.3 (*14.4%*)	39.9 (*32.4%*)	41.0 (*32.2%*)	3.5 (*0.63%*)	4.3 (*0.75%*)
	24 to 35 mo	1200	1182	159.8 (*53.3%*)	165.5 (*56.0%*)	46.2 (*15.4%*)	45.0 (*15.2%*)	43.9 * (*32.9%*)	**39.4** (*30.0%*)	5.7 (*0.95%*)	5.9 (*1.0%*)
Taylor et al. [14] ^f^	3.5 y	*1350* ^g^	*1363*	175 (*51.9%*) ^g^	178 (*52.2%*)	46.1 (*13.7%*) ^g^	47.9 (*14.1%*)	55.9 (*37.3%*) ^g^	55.2 (*36.5%*)	--	--
		*1346* ^h^	*1,363*	176 (*52.3%*) ^h^	178 (*52.2%*)	44.7 * (*13.3%)*^h^	**47.9** (*14.1%*)	56.9 (*38.1%*) ^h^	55.2 (*36.5%*)		
Antoniou et al. [24]	9 y	(*329*)	(*324*)	--	--	--	--	--	--	--	--
Xue et al. [16]	7 to 12 y	1297 *	**1470**	169 * (*52.1%*)	**198** (*53.9%*)	46.2 * *(14.3%)*	**54.9** *(14.9%)*	49.6 *(34.4%*)	56.9 (*34.8%*)	5.03 * ***(0.78%)***	**6.37** **(*0.87%*)**
Xue et al. [17]	3 to 7 y	1554	1628	214 (*55.1%*)	225 (*55.3%*)	51.8 * *(13.3%)*	**55.8** *(13.7%*)	57.8 (*33.5%*)	59.3 (32.8%)	6.8 (*0.88%*)	7.6 (*0.93%*)
Cardona Cano et al. [25]	14 mo	1293 *	**1329**	--	--	--	--	--	--	--	--
Tharner et al. [30]	14 mo	1300	1316	--	--	--	--	--	--	--	--
Dubois et al. [37,38]	4.5 y	1548 *	**1625**	212 (54.8) *	219 **(53.6%)**	52.5 * (13.4%) *	**58.6** **(14.2%)**	54.5 * (31.9%)	**57.5** (33.3%)	--	--
Galloway et al. [40]	9 y	1778	1838	*248* (55.7%)	*262* (57.1%)	*62* (14.0%)	*63* (13.7%)	*63* (31.7%)	*65* (31.9%)	11.2 * (*1.26%*)	**12.7** (*1.38%*)
Carruth et al. [20]	7 to 8 mo	785 *	**838**	**108** (55%)	**119** (56%)	*14.2* (*7.3%*)	*16.3* (*7.8%*)	33 (37%)	33 (36%)	--	--
	9 to 11 mo	911 *	**989**	130 (57%)	138 (56%)	*19.0* (*8.3%*)	*23.8* *(9.6%)*	35 * (34%)	**38** (35%)	--	--
Jacobi et al. [5]	3.5 y ^i^	1559	1546	--	--	--	--	--	--	--	--
	5.5 y ^i^	1589 *	**1659**	--	--	--	--	--	--	--	--
Carruth and Skinner [3] ^j^	34 mo	1468 ± 318	1300 ^j^	--	--	49 ± 14 *(13.3%)*	16 ^j^	53 ± 15 *(32.5%)*	--	--	--
	42 mo	1380 ± 261	1300 ^j^	--	--	46 ± 14 *(13.3%)*	16 ^j^	45 ± 13 *(29.3%)*	--	--	--
	60 mo	1716 ± 426	1800 ^j^	--	--	56 ± 18 *(13.1%)*	24 ^j^	61 ± 21 *(32.0%)*	--	--	--
	72 mo	1762 ± 388	1800 ^j^	--	--	60 ± 17 *(13.6%)*	24 ^j^	62 ± 15 *(31.7%)*	--	--	--
	84 mo	1812 ± 338	1800 ^j^	--	--	58 ± 18 *(12.8%)*	24 ^j^	64 ± 16 *(31.8%)*	--	--	--
Carruth et al. [9]	24 to 36 mo	1468	1472	*198.8* *(54.2%)*	*196.8* *(53.5%)*	49 *(13.3%)*	52 *(14.1%)*	53 *(32.5%)*	53 *(32.4%)*	--	--

Abbreviations: β = regression coefficient; BW = body weight; CI = confidence intervals; d = day; mo = months; NPE = non-picky eater(s); PE = picky eater(s); y = years; %E = percentage of total energy. ^a^ Values are means, unless otherwise indicated. Values appearing in bold-type font and with an asterisk (*) are significantly different between PE and NPE. Values in italics were calculated using information provided in the publication, as well as the standard Atwater factors of 4, 4 and 9 kcal per gram of carbohydrate, protein and fat, respectively. For dietary fiber, a caloric value of 2 kcal per gram was used. If dietary fiber was not reported and levels of some of the macronutrients were missing, then these were estimated assuming dietary fiber was 0 g/d. ^b^ Values reported are coefficients from adjusted linear regression models (adjusted for baseline measures of outcome; group intervention/control, age, sex, and maternal education) (β, 95% CI), in order to examine the influence of pickiness on nutrient energy intake (kJ) and macronutrients (% E), after 15 mo follow-up. Only results for PE and not “a little picky” are presented in this table. ^c^ The energy and macronutrient intakes of children with four PE behaviors relative to NPE children were studied by the authors (i.e., eating small amount, refusal to eat specific food groups, neophobic behavior, and preference for a specific food preparation method). The data presented in the table are related to the first two PE behaviors. ^d^ The classification of PE was based on “eating small amounts”. ^e^ The classification of PE was based on the refusal of ≥2 food groups. ^f^ PE status was determined when the children were 3 y, and dietary intakes were assessed when the children were 3.5 y. ^g^ Picky eating score 1; quite choosy (assessed with the use of a questionnaire). ^h^ Picky eating score 2; very choosy (assessed with the use of a questionnaire). ^i^ There was a significant effect of picky eater status × gender × time in this experimental study where the food intake of children was assessed over two separate 24-hour periods. Amongst the boys, PE and NPE increased their 24-hour caloric intake between ages 3.5 and 5.5. Amongst the girls, NPE also increased their 24-hour caloric intake, whereas PE decreased their caloric intake, between ages 3.5 and 5.5. j Only intakes for children perceived as PE were reported by Carruth and Skinner [3]. For comparison, the dietary recommendations for the intakes of energy and protein (but not fat), as reported by Carruth and Skinner [3], are presented in this Table instead under the column for NPE. The values reported by Carruth et al. [9] for PE (24 to 36 mo) are identical to those reported by Carruth and Skinner [3] for PE (34 mo.), however, intakes for NPE at 24 to 36 mo were reported only by Carruth et al. [9], and so the two studies are reported separately in the table.

**Table 6:** Intakes of Vitamins A, D, E and C in PE and NPE ^a,b^.

Reference	Age at Intake Analysis	Vitamin A (μg RAE/d)			Vitamin D (μg/d)			Vitamin E (mg/d)			Vitamin C (mg/d)		
		PE	NPE	RDA/AI	PE	NPE	RDA/AI	PE	NPE	RDA/AI	PE	NPE	RDA/AI
Li et al. [12]	6 to 11 mo	509 ± 385 *	**670 ± 564**	400	--	--	--	7.0 ± 5.5	7.6 ± 9.2	3	45 ± 43 *	**58 ± 45**	50
	12 to 23 mo	908 ± 802	867 ± 801	500	--	--	--	11.1 ± 7.7	12.5 ± 8.7	4	88 ± 86	87 ± 93	60
	24 to 35 mo	713 ± 629	691 ± 553	500	--	--	--	12.4 ± 8.3	12.4 ± 9.2	4	88 ± 290	69 ± 68	60
Kwon et al. [22] ^c,d^	1 to 5 y	393 ± 205 *	**460 ± 239**	300 to 400	--	--	--	--	--	--	77 ± 55 *	**94 ± 61**	15 to 25
Taylor et al. [14] ^d,e^	3.5 y	365 (332, 399)	370 (331, 409)	300 to 400	1.7 (1.4, 2.1)	1.8 (1.7, 1.9)	15	5.7 (5.3, 6.1)	6.2 (5.9, 6.4)	6 to 7	54 (46, 62)	55 (51, 59)	15 to 25
Xue et al. [16] ^d^	7 to 12 y	229 * **(115, 378)**	**294** **(165, 430)**	400 to 600	--	--	--	16.4 * **(11.6,23.6)**	**19.3** **(12.8, 26.3)**	7 to 11	36 * **(17, 68)**	**53** **(28, 100)**	25 to 45
Xue et al. [17] ^d^	3 to 7 y	543 ± 43	482 ± 27	300 to 400	--	--	--	19.1 ± 0.6	19.1 ± 0.5	6 to 7	67 ± 3	64 ± 2	15 to 25
Galloway et al. [39]	9 yrs	669.7 ± 282	718.1 ± 288	600	--	--	--	5.6 ± 1.5 *	**6.8 ± 2.3**	11	67.0 ± 39	78.0 ± 39	45
Carruth et al. [20]	7 to 8 mo	--	--	--	--	--	--	9.3 ± 3.0 *	**11.2 ± 8.7**	6	96 ± 43 *	**107 ± 48**	50
	9 to 11 mo	--	--	--	--	--	--	9.4 ± 4.0	9.6 ± 4.2	6	103 ± 52	105 ± 56	50
Carruth and Skinner [3] ^f^	34 mo	754 ± 528	--	400	4.0 ± 2.6	--	5	4.0 ± 2.8	--	6	88 ± 74	--	15
	42 mo	505 ± 217	--	400	3.9 ± 2.2	--	5	2.8 ± 2.0	--	6	67 ± 49	--	15
	60 mo	751 ± 372	--	500	4.3 ± 2.3	--	5	2.8 ± 2.2	--	7	68 ± 38	--	25
	72 mo	766 ± 459	--	500	4.5 ± 2.3	--	5	4.2 ± 2.9	--	7	75 ± 39	--	25
	84 mo	718 ± 442	--	500	4.8 ± 2.6	--	5	4.7 ± 3.9	--	7	88 ± 54	--	25
Carruth et al. [9]	24 to 36 mo	754 ± 528	780 ± 466	400	4.0 ± 2.6	4.8 ± 2.9	10	4.0 ± 2.8	4.5 ± 3.3	6	88 ± 74	93 ± 66	40

Abbreviations: AI = Adequate Intake; CI = confidence intervals; d = day; DRI = dietary reference intake; mo = months; NPE = non-picky eater(s); PE = picky eater(s); RAE = retinal activity equivalents; RDA = recommended dietary allowance; SD = standard deviation; SEM = standard error of the mean; U.S. = United States; y = years. ^a^ Values are mean ± SD, mean ± SEM, median (25th, 75th percentiles), or median (95% CI), as reported in the publications. ^b^ Values appearing in bold-type font and with an asterisk (*) are those for which a statistically significant difference between PE and NPE were reported by the study authors. ^c^ Of the four PE behavior constructs studied, the results for “Eating small amounts” are presented; that is, for children whose mean score of responses was >3 were classified as “PE”. ^d^ The RDA/AI for the nutrients were NR in the publication; therefore, the U.S. DRIs have been used for the appropriate age range. ^e^ Dietary intakes assessed at 3.5 y are presented for children categorized as PE or NPE at 3 y; values presented are for the comparison of the “not choosy” group *versus* “very choosy”. Dietary intakes of PE and NPE at 7.5 y are not reported in this table, as PE status was assessed 2 y earlier. ^f^ Intakes for children perceived as PE were reported in the publication by Carruth and Skinner [3], while Carruth et al. [9] additionally reported values for NPE; however, the RDA for vitamin C was reported as 40 mg/d by Carruth et al. [9] and as 15 mg/d by Carruth and Skinner [3]; thus, the two studies are reported separately in the table.

**Table 7:** Intakes of B Vitamins in PE and NPE ^a,b^.

Reference	Age at Intake Analysis	Folate (μg/d)			Vitamin B6 (mg/d)			Vitamin B12 (μg/d)			Thiamine (mg/d)			Riboflavin (mg/d)			Niacin (mg/d)		
		PE	NPE	RDA/AI	PE	NPE	RDA/AI	PE	NPE	RDA/AI	PE	NPE	RDA/AI	PE	NPE	RDA/AI	PE	NPE	RDA/AI
Li et al. [12]	6 to 11 mo	82 ± 124	92 ± 89	80 ^d^	0.2 ± 0.3 *	**0.3 ± 0.4**	0.3 ^d^	--	--	--	--	--	--	0.6 ± 0.5 *	**0.8 ± 0.6**	0.5	4.6 ± 4.2	4.2 ± 3.3	3
	12 to 23 mo	141 ± 110	138 ± 108	120 ^d^	0.6 ± 0.8	0.5 ± 0.5	0.4 ^d^	--	--	--	0.4 ± 0.8	0.4 ± 0.7	0.6	1.2 ± 1.2	1.2 ± 1.2	0.6	6.8 ± 4.4	7.7 ± 5.8	6
	24 to 35 mo	178 ± 196	173 ± 170	120 ^d^	0.5 ± 0.4	0.6 ± 0.8	0.4 ^d^	--	--	--	0.2 ± 0.4	0.3 ± 0.8	0.6	1.0 ± 0.8	1.1 ± 1.1	0.6	8.6 ± 5.3	8.9 ± 6.6	6
Kwon et al. [22] ^c,d^	1 to 5 y	--	--	--	--	--	--	--	--	--	0.66 ± 0.24 *	**0.78 ± 0.26**	0.5 to 0.6	0.9 ± 0.3 *	**1.0 ± 0.3**	0.5 to 0.6	8.0 ± 3.0 *	**9.0 ± 3.0**	6 to 8
Taylor et al. [14] ^d,e^	3.5 y	146 (138, 154)	154 (150, 159)	150	1.2 * **(1.1, 1.3)**	**1.3** **(1.3, 1.4)**	0.5	3.1 (2.9, 3.3)	3.1 (3.0, 3.3)	0.9	0.9 (0.9, 1.0)	1.0 (0.9, 1.0)	0.5	1.5 (1.4, 1.6)	1.4 (1.4, 1.5)	0.5	19.4 * **(18.6, 20.2)**	**21.2** **(20.7, 21.8)**	6
Xue et al. [16] ^d^	7 to 12 y	--	--	--	--	--	--	--	---	--	0.5 * **(0.3, 0.8)**	**0.6** **(0.4, 0.9)**	0.6 to 0.9	0.7 * **(0.4, 0.9)**	**0.8** **(0.5, 1.1)**	0.6 to 0.9	9.2 (5.5, 13.5)	11.3 (6.8, 16.4)	8 to 12
Xue et al. [17] ^d^	3 to 7 y	--	--	--	--	--	--	--	--	--	0.9 ± 0.1	0.8 ± 0.0	0.5 to 0.6	0.9 ± 0.1	0.9 ± 0.1	0.5 to 0.6	11.0 ± 0.3	11.2 ± 0.3	6 to 8
Galloway et al. [39]	9 y	303 ± 92 *	**330 ± 87**	300	1.5 ± 0.49	1.4 ± 0.41	1.0	--	--	--	--	--	--	--	--	--	--	--	--
Carruth et al. [20]	7 to 8 mo	156 ± 88	190 ± 335	80	0.7 ± 0.4	0.7 ± 0.4	0.3	1.6 ± 1.1	1.7 ± 1.4	0.5	0.73 ± 0.3 *	**0.84 ± 0.4**	0.3	1.1 ± 0.5 *	**1.2 ± 0.6**	0.4	9 ± 4 *	**10 ± 5**	4
	9 to 11 mo	199 ± 103 *	**228 ± 141**	80	0.8 ± 0.4 *	**0.9 ± 0.5**	0.3	1.9 ± 1.1 *	**2.2 ± 1.0**	0.5	0.88 ± 0.4 *	**0.94 ± 0.4**	0.3	1.3 ± 0.5 *	**1.4 ± 0.6**	0.4	11 ± 5	11 ± 5	4
Carruth and Skinner [3] ^f^	34 mo	129 ± 63	--	150	1.1 ± 0.4	--	0.5	2.8 ± 1.4	--	0.9	--	--	--	--	--	--	--	--	--
	42 mo	153 ± 60	--	150	1.1 ± 0.4	--	0.5	2.9 ± 1.4	--	0.9	--	--	--	--	--	--	--	--	--
	60 mo	172 ± 52	--	200	1.3 ± 0.4	--	0.6	3.7 ± 1.4	--	1.2	--	--	--	--	--	--	--	--	--
	72 mo	200 ± 83	--	200	1.5 ± 0.6	--	0.6	4.0 ± 1.7	--	1.2	--	--	--	--	--	--	--	--	--
	84 mo	202 ± 131	--	200	1.5 ± 0.6	--	0.6	3.7 ± 1.8	--	1.2	--	--	--	--	--	--	--	--	--
Carruth et al. [9]	24 to 36 mo	129 ± 63	158 ± 87	50	1.1 ± 0.4	1.1 ± 0.4	1.0	2.8 ± 1.4	3.0 ± 1.4	0.7	--	--	--	--	--	--	--	--	--

Abbreviations: AI = Adequate Intake; CI = confidence intervals; d = day; DRI = dietary reference intake; mo = months; NPE = non-picky eater(s); PE = picky eater(s); RDA = recommended dietary allowance; SD = standard deviation; SEM = standard error of the mean; U.S. = United States; y = years. ^a^ Values are mean±SD, mean±SEM, median (25^th^, 75^th^ percentiles), or median (95% CI), as reported in the publications. ^b^ Values appearing in bold-type font and with an asterisk (*) are those for which a statistically significant difference between PE and NPE were reported by the study authors. ^c^ Of the four PE behavior constructs studied, the results for “Eating small amounts” are presented; that is, for children whose mean score of responses was >3 were classified as “PE”. ^d^ The RDA/AI for the nutrients were not reported in the publication; therefore, the U.S. DRIs have been used for the appropriate age range. ^e^ Dietary intakes assessed at 3.5 y are presented for children categorized as PE or NPE at 3 y; values presented are for the comparison of the “not choosy” group versus “very choosy”. Dietary intakes of PE and NPE at 7.5 y are not reported in this table, as PE status was assessed 2 years earlier. ^f^ Intakes for children perceived as PE were reported in the publication by Carruth and Skinner [3], while Carruth et al. [9] additionally reported values for NPE; however, the RDAs for folate and vitamins B6 and B12 were reported by Carruth et al. [9] as 50 μg/d, 1 mg/d, and 0.7 μg/d, respectively for children aged 24 to 36 mo, but were reported as 150 μg/d, 0.5 mg/d, and 0.9 μg/d, respectively, by Carruth and Skinner [3]; thus, the two studies are reported separately in the table.

**Table 8:** Intakes of Minerals in PE and NPE ^a,b^.

Reference	Age at Intake Analysis	Calcium (mg/d)			Iron (mg/d)			Magnesium (mg/d)			Zinc (mg/d)		
		PE	NPE	RDA/AI	PE	NPE	RDA/AI	PE	NPE	RDA/AI	PE	NPE	RDA/AI
Li et al. [12]	6 to 11 mo	503 ± 557	539 ± 408	400	8.3 ± 6.6	8.7 ± 6.0	10	115 ± 69	122 ± 81	70	5.3 ± 4.6	5.0 ± 3.1	8
	12 to 23 mo	812 ± 736	801 ± 853	600	13.0 ± 9.0	13.4 ± 9.5	12	144 ± 79	154 ± 99	100	7.5 ± 5.1	7.7 ± 5.6	9
	24 to 35 mo	650 ± 516	642 ± 536	600	15.0 ± 11.3	15.3 ± 15.1	12	156 ± 77	162 ± 93	100	7.7 ± 4.2	7.6 ± 4.5	8
Kwon et al. [22] ^c,d^	1 to 5 y	416 ± 146	449 ± 217	700 to 1000	8 ± 3 *	**10 ± 4**	7 to 10	--	--	--	--	--	--
Taylor et al. [14] ^e^	3.5 y	796 (740, 853)	754(728, 780)	350	5.9 (5.5, 6.2) *	**6.5 (6.3, 6.6)**	6.9	--	--	--	4.9 (4.6, 5.1) *	**5.3 (5.2, 5.5)**	5
Xue et al. [16] ^d^	7 to 12 y	289 (157, 471) *	**330(193, 545)**	1000 to 1300	12.6 (9, 18) *	**14.7 (11, 22)**	8 to 10	173 (121, 234) *	**209 (151, 284)**	130 to 240	6.6 (4.6, 8.9) *	**7.5 (5.5, 10.6)**	5 to 8
Xue et al. [17] ^d^	3 to 7 y	446 ± 24	443 ± 23	700 to 1000	15.7 ± 0.4*	**17.3 ± 0.5**	7 to 10	210 ± 5	230 ± 7	80 to 130	8.3 ± 0.2 *	**9.3 ± 0.4**	3 to 5
Galloway et al. [39]	9 y	911 ± 320	905 ± 288	1300	12.0 ± 3.9	12.9 ± 3.8	8	212.3 ± 47.3	213.7 ± 52.7	240	8.7 ± 2.6	9.0 ± 2.6	8
Carruth et al. [20]	7 to 8 mo	542 ± 241	597 ± 235	270	14 ± 9 *	**17 ± 8**	11	98 ± 43	105 ± 40	75	5 ± 2	5 ± 2	3
	9 to 11 mo	608 ± 252 *	**693 ± 338**	270	15 ± 10	16 ± 9	11	115 ± 44 *	**131 ± 60**	75	5 ± 2 *	**6 ± 3**	3
Carruth and Skinner [3] ^f^	34 mo	763 ± 343	--	500	9 ± 4	--	10	157 ± 49	--	80	6 ± 3	--	10
	42 mo	714 ± 242	--	500	9 ± 3	--	10	158 ± 47	--	80	7 ± 3	--	10
	60 mo	911 ± 344	--	800	10 ± 3	--	10	192 ± 66	--	130	8 ± 3	--	10
	72 mo	878 ± 308	--	800	13 ± 4	--	10	202 ± 59	--	130	9 ± 2	--	10
	84 mo	888 ± 384	--	800	12 ± 5	--	10	196 ± 63	--	130	8 ± 3	--	10
Carruth et al. [9]	24 to 36 mo	763 ± 343	853 ± 347	800	9 ± 4	10 ± 7	10	157 ± 49	167 ± 57	80	6 ± 3	6 ± 3	10

Abbreviations: AI = Adequate Intake; CI = confidence intervals; d = day; DRI = dietary reference intake; mo = months; NR = not reported; NPE = non-picky eater(s); PE = picky eater(s); RDA = recommended dietary allowance; RNI = Reference Nutrient Intake; SD = standard deviation; SEM = standard error of the mean; U.S. = United States; y = years. ^a^ Values are mean±SD, mean ± SEM, median (25^th^, 75^th^ percentiles), or median (95% CI), as reported in the publications. ^b^ Values appearing in bold-type font and with an asterisk (*) are those for which a statistically significant difference between PE and NPE were reported by the study authors. ^c^ Of the four PE behavior constructs studied, the results for “Eating small amounts” are presented; that is, for children whose mean score of responses was >3 were classified as “PE”. ^d^ The RDA/AI for the nutrients were NR in the publication; therefore, the U.S. DRIs have been used for the appropriate age range. ^e^ Dietary intakes assessed at 3.5 y are presented for children categorized as PE or NPE at 3 y; values presented are for the comparison of the “not choosy” group versus the “very choosy” group. Dietary intakes of PE and NPE at 7.5 y are not reported in this table, as PE status was assessed 2 years earlier. Significantly lower intakes of selenium among PE (38.3 mg/d) versus NPE (43.6 mg/d) were reported. In terms of the % children with intakes below the recommended intakes for iron and zinc, one-half to three-fourths of both groups of children had intakes below the RNI; however, a significantly larger number of PE had intakes of iron below the recommended intakes when compared to NPE. ^f^ Intakes for children perceived as PE were reported in the publication by Carruth and Skinner [3], while Carruth et al. [9] additionally reported values for NPE; however, the RDA for calcium was reported by Carruth et al. [9] as 800 mg/d for children aged 24 to 36 mo, but was reported as 500 mg/d by Carruth and Skinner [3]; thus, the two studies are reported separately in the table.

**Table 9:** Body weight, height, and growth status of children classified as PE versus NPE.

Reference	Age at BL	Sample Size (%PE)	Outcome(s) Assessed	Results		Between Group Statistical Significance
				PE	NPE	
**Studies where PE status was not significantly associated with impaired growth**						
Li et al. [12]	6 to 35 mo	*n* = 1414 (23.8%)	Proportion of children OW [*n* (%)], defined as a weight-for height z-score >2	47 (14.2%)	169 (16.0%)	NS
			Proportion of children NW [*n* (%)], defined as weight-for height z-score between −2 and 2	278 (84.2%)	873 (83.0%)	NS
			Proportion of children UW [*n* (%)], defined as weight-for height z-score < 2	6 (1.6%)	11 (1.0%)	NS
Rohde et al. [13]	2 to 6 y	*n* = 271 (16%)	BL BMI z-score [mean (95% CI)]	0.1 (−1.3;1.2)	0.1 (−1.2;1.1)	NS
			15-mo follow-up BMI z-score [mean (95% CI)]	0.04 (−0.13; 0.21)	--	NS
Werthmann et al. [28]	2.5 to 4 y	*n* = 32 (NR)	BMI	--	--	NS
Equit et al. [31]	4 to 7 y	*n* = 1090 (34%)	Proportion UW, defined as BMI ≤ 3rd percentile	4.0%	2.8%	NS
van der Horst [33]	6 to 12 y	*n* = 305 (45%)	Proportion UW (internationally-based BMI cut-offs)	7.2%	9.9%	NS
Mascola et al. [7]	11 y	*n* = 120 (22%)	BMI	--	--	NS
Jacobi et al. [1]	7.7 to 12.7 y	*n* = 426 (19%)	BMI (mean ± SD)	17.16 ± 2.62	17.67 ± 3.01	NS
Lewinsohn et al. [41]	3 y	*n* = 93 (NR)	BMI	--	--	NS
Jacobi et al. [5]	3.5 to 5.5 y	*n* = 135 (21%)	BMI (mean ± SD)	Age 4 y: 15.8 ± 1.2 Age 5 y: 15.9 ± 1.2	Age 4 y: 16.4 ± 1.4 Age 5 y: 16.3 ± 1.4	NS NS
Carruth and Skinner [3]	34 to 84 mo	*n* = 71 (30 to 49%, based on age)	BW	--	--	NS
			Body height	--	--	NS
Carruth et al. [9]	24 to 36 mo	*n* = 118 (36%)	BW, in kg (mean ± SD)	M: 13.3 ± 1.5 F: 12.5 ± 1.4	M: 13.5 ± 1.4 F: 12.5 ± 1.9	NS NS
			Body height, in cm (mean ± SD)	M: 88.9 ± 3.8 F: 88.1 ± 3.0	M: 89.4 ± 3.8 F: 86.4 ± 4.1	NS NS
Rydell et al. [42]	6 to 11 y	*n* = 240 (6 to 16%, based on PE status at school, home, and school + home)	Proportion of children with weight:height score of −1 SD	Home and school choosy: 26% School-choosy: 14% Home-choosy: 7%	Not choosy: 13%	NS
**Studies where PE status was associated with significantly impaired growth in some (but not all) of the parameters assessed**						
Kwon et al. [22]	1 to 5 y	*n* = 184 (70.1%, overall; 29.9%, eating small amount; 44.0%, refusal to eat specific food groups)	***Children classified as PE based on “eating small amounts”*^a^**			
			Weight-for-age z-score	−0.2 ± 0.9	0.2 ± 0.8	SS ↓ in PE
			Height-for-age z-score	−0.5 ± 1.1	−0.2 ± 1.1	NS
			BMI-for-age z-score	0.0 ± 1.3	0.4 ± 0.9	SS ↓ in PE
			***Children who refused*** ***≥2 food groups were classified as PE for “refusal to eat specific food groups”*^b^**			
			Weight-for-age z-score	0.0 ± 0.9	0.1 ± 0.7	NS
			Height-for-age z-score	−0.3 ± 1.1	−0.2 ± 1.1	NS
			BMI-for-age z-score	0.2 ± 0.9	0.3 ± 1.1	NS
Antoniou et al. [24]	5 y	*n* = 1024 (39.3%)	Height, in cm (mean ± SD)	111.14 ± 6.15	112.58 ± 6.06	SS ↓ in PE
			BMI, in kg/m^2^ (mean ± SD)	15.14 ± 1.37	15.36 ± 1.40	SS ↓ in PE
			UW [*n* (%)]	86 (22.87%)	104 (17.75%)	SS ↑ in PE
			NW [*n* (%)]	269 (71.54%)	425 (72.53%)	SS ↓ in PE
			OW/OB [*n* (%)]	21 (5.59%)	57 (9.73%)	SS ↓ in PE
			Change in BMI from BL until 9 y in children at risk of becoming UW and with low NW status at BL [adjusted β (95% CI)]	+0.05 (−0.11; +0.22)	NA	NS
			Change in BMI from BL until 9 y in children at risk of becoming OW & with high NW status at BL [adjusted β (95% CI)]	−0.08 (−0.25; +0.10)	NA	NS
Berger et al. [23]	5 to 15 y	*n* = 197 (18% persistent PE)	BMI z-scores	--	--	SS ↓ in persistent PE (at all time points, BMI tracked along the 50th percentile in PE and along the 65th percentile in NPE)
			Prevalence of UW	--	--	NS
			Prevalence of OW/OB, defined as BMI ≥85th percentile	<2%	5 y: 21% 7 y: 22% 9 y: 36% 11 y: 34% 13 y: 28% 15 y: 24%	--
Xue et al. [17]	3 to 7 y	*n* = 937 (54%)	BW, in kg (mean ± SEM)	18.11 ± 0.13	18.96 ± 0.16	SS ↓ in PE
			Weight for age (mean ± SEM)	0.08 ± 0.04	0.23 ± 0.05	SS ↓ in PE
			Height, in cm (mean ± SEM)	108.66 ± 0.33	110.45 ± 0.39	NS
			Height for age (mean ± SEM)	0.18 ± 0.04	0.31 ± 0.05	NS
			BMI, in kg/m^2^ (mean ± SEM)	15.28 ± 0.06	15.46 ± 0.07	SS ↓ in PE
			BMI for age (mean ± SEM)	−0.06 ± 0.04	0.04 ± 0.05	NS
Ekstein et al. [34]	14 to 91 mo	*n* = 170 (NR)	Proportion UW (weight-for-length below 5^th^ percentile)	20.6%	6.6%	Odds of being UW was SS ↑ in PE vs. NPE [adjusted OR = 3.6 (1.2 to 10.7)] ^c^
			BW, in kg (mean ± SD)	13.3 ± 4.3	14.1 ± 5.1	NS
Wright et al. [6]	30 mo	*n* = 455 (8%, definitely faddy; 15%, maybe faddy; 20%, eating problem)	z-score for BW (mean ± SD)	Maybe faddy: 0.2 ± 1.3 Definitely faddy: 0.04 ± 1.1	Not faddy: 0.5 ± 1.2	NS
				Eating problem: 0.1 ± 1.3	No eating problem: 0.5 ± 1.1	SS ↓ in PE
			z-score for body height (mean ± SD)	Maybe faddy: 0.2 ± 1.4 Definitely faddy: −0.2 ± 1.1	Not faddy: 0.3 ± 1.0	NS
				Eating problem: −0.2 ± 1.3	No eating problem: 0.3 ± 1.0	SS ↓ in PE
			Thrive Index (i.e., measure of weight gain starting from birth)	Maybe faddy: 0.3 ± 1.2 Definitely faddy: 0.1 ± 1.0	Not faddy: 0.5 ± 1.1	NS
				Eating problem: 0.1 ± 1.2	No eating problem: 0.5 ± 1.1	SS ↓weight gained since birth in PE
Chatoor et al. [21]	12 to 37 mo	*n* = 68 (NR)	% Ideal BW	102.4%	107.7%	SS ↓ in PE but PE status was not a predictor of % ideal BW in multiple regression analysis.
**Studies where PE status was associated with significantly impaired growth in all of the parameters assessed**						
de Barse et al. [26]	4 y	*n* = 4191 (5.7%)	BMI-SDS [β (95% CI)]	−0.37 (−0.47, −0.26)	NA	SS ↓ in PE
			Fat Mass Index SDS [β (95% CI)]	−0.22 (−0.33, −0.12)	NA	SS ↓ in PE
			FFM Index SDS [β (95% CI)]	−0.41 (−0.54, −0.29)	NA	SS ↓ in PE
			Change in BMI SDS from 4 to 6 y, adjusting for BL BMI at 4 y	0.11 lower BMI-SDS at 6 y (95 % CI: −0.19, −0.04)	NA	SS ↓ in PE (due mainly to a decrease in FFM)
			Risk of becoming UW [OR (95% CI)]	2.28 (1.34, 3.87)	NA	SS ↑ in PE
Rodenburg et al. [32]	7 to 10 y	*n* = 1275 (NR)	Child BMI z-score in 2009 (at time of PE assessment)—adjusted β (P-value) ^d^	−0.08 (P < 0.01)	NA	SS ↓ with ↑ food fussiness
			Child BMI z-score in 2010 (1 y after PE assessment)—adjusted β (P-value) ^e^	−0.08 (P < 0.01)	NA	SS ↓ with ↑ food fussiness
Xue et al. [16]	7 to 12 y	*n* = 793 (59.3%)	Height, in cm	135.0 ± 11.2	138.0 ± 11.2	SS ↓ in PE
			Height-for-age z-score	0.13 ± 1.03	0.29 ± 1.16	SS ↓ in PE
			BW, in kg	31.0 ± 9.6	34.8 ± 11.4	SS ↓ in PE
			Weight-for-age z-score	−0.07 ± 1.09	0.25 ± 1.17	SS ↓ in PE
			BMI, in kg/m^2^	16.7 ± 3.0	17.9 ± 3.9	SS ↓ in PE
			BMI-for-age z-score	0.09 ± 1.54	0.09 ± 1.54	SS ↓ in PE
Tharner et al. [30]	4 y	*n* = 4915 (5.6%)	BMI, in kg/m^2^ (mean ± SEM)	15.45 ± 0.09	15.84 ± 0.03	SS ↓ in PE
			Proportion UW (internationally-based BMI cut-offs)	19.3%	12.3%	SS ↑ in PE
Dubois et al. [37]	2.5 y	*n* = 1498 (14 to 16.9% at each age; 5.5% at all 3 time points)	Proportion UW (BMI < 10^th^ percentile)	PE at 1 or 2 time points: 18.3%	Never PE: 13.2%	NS
				PE at 3 time points: 26.8%	Never PE: 13.2%	Odds of being UW was SS ↑ for PE vs. NPE [adjusted OR = 2.42 (1.38–4.22)] ^f^
			Proportion OW (BMI ≥ 95^th^ percentile)	PE at 1 or 2 time points: 6.9%	Never PE: 9.9%	NS
				PE at 3 time points: 7.7%	Never PE: 9.9%	NS
Galloway et al. [39]	9 y	*n* = 173 (48%)	BMI, in kg/m^2^ (mean ± SD)	17.9 ± 2.7	18.9 ± 3.4	SS ↓ in PE
			% Body Fat (mean ± SD)	25.6 ± 6.6%	27.8 ± 7.4%	SS ↓ in PE
			Proportion OW (BMI > 85^th^ percentile) or OB (BMI > 95^th^ percentile)	18%	43%	SS ↓ in PE
Carruth et al. [20]	4 to 24 mo	*n* = 2854 (19 to 50%, based on age)	Odds of being a PE according to weight-for-age percentiles	--	--	Odds of being a PE were SS ↓ in children with higher weight-for-age percentiles: 0 to 25^th^: OR = 1.00 25 to 50^th^: OR = 0.62 (0.45 to 0.86) 50 to 75^th^: OR = 0.61 (0.45 to 0.84) 75 to 100^th^: OR = 0.66 (0.49 to 0.88)

Abbreviations: ↑ = greater; ↓ = lower; β = regression coefficient; BL = baseline; BMI = body mass index; BW = body weight; CI = confidence intervals; F = females; FFM = fat-free mass; M = males; mo = months; *n* = number; NA = not applicable; NPE = non-picky eater(s); NR = not reported; NS = not significant; NW = normal weight; OB = obese; OR = odds ratio; OW = overweight; PE = picky eater(s); SD = standard deviation; SDS = standard deviation score; SEM = standard error of the mean; SS = statistically significant; UW = underweight; y = years. ^a^ “Eating small amounts” was a summated rating scale. Children whose mean score of response was >3 were classified as PE for “eating small amounts”. ^b^ “Refusal to eat specific food groups” was evaluated based on the number of food groups refused. The cut-off number was set based on the mean number of food groups with responses more than neutral (1.8 for refused food groups). Children who refused two or more food groups were classified as PE for “refusal to eat specific food groups”. ^c^ Although not explicitly stated in the publication, it appears the OR may have been adjusted for gender and age. ^d^ Adjusted for age, gender, socioeconomic status, ethnicity, and parenteral BMI in 2009. ^e^ Adjusted for age, gender, socioeconomic status, ethnicity, and parenteral BMI in 2010. ^f^ Adjusted for all the included behaviors and for child sex and birth weight, income level, parental overweight/obesity, and mother’s smoking status during pregnancy.
